# Small molecular weight polyfluoroalkyl phosphonates induce ROS-mediated cytotoxicity in glioblastoma cells: a molecular mechanism study

**DOI:** 10.1038/s41598-025-22754-0

**Published:** 2025-11-06

**Authors:** Patryk Wołodkiewicz, Michał Juszczak, Paweł Tokarz, Katarzyna Woźniak, Paulina Tokarz

**Affiliations:** 1 Faculty of Biology and Environmental Protection, Department of Molecular Genetics, University of Lodz, Pomorska 141/143, 90-236 Lodz, Poland; 2 University of Lodz, University of Lodz Doctoral School of Exact and Natural Sciences, Matejki 21/23, 90-237 Lodz, Poland; 3 Faculty of Chemistry, Laboratory of Molecular Spectroscopy, University of Lodz, Tamka 12, 91-403 Lodz, Poland

**Keywords:** Apoptosis, Cell cycle arrest, Cytotoxicity, DNA damage, Glioblastoma, ROS, ZOT, Biochemistry, Cancer, Cell biology, Drug discovery, Oncology

## Abstract

Glioblastoma (GBM) is an aggressive brain tumour with limited treatment options and poor patient survival, largely due to the blood-brain barrier (BBB) restricting effective drug delivery. In this study, we focused on two small molecular weight polyfluoroalkyl phosphonates, ZOT_5_-1-Me and ZOT_5_-1-Et, designed to permeate the BBB. Comprehensive in vitro analyses using U-87 MG cells and a panel of glioma cell lines revealed that both compounds exhibit potent cytostatic and cytotoxic activities. Mechanistically, they induce reactive oxygen species (ROS) production, triggering both intrinsic and extrinsic apoptotic pathways via caspase-dependent and caspase-independent mechanisms. Additionally, ZOT_5_-1-Me and ZOT_5_-1-Et induced DNA damage, including single-strand breaks and alkali-labile sites. Notably, ZOT_5_-1-Me also caused significant DNA double-strand breaks and impaired DNA repair. Furthermore, both compounds exhibited antiproliferative effects by inducing cell cycle arrest in the S phase and activating p53-p21 signalling pathway. Pre-treatment with the ROS scavenger N-acetyl-L-cysteine (NAC) effectively abrogated these cytotoxic effects, underscoring the central role of oxidative stress in mediating the compounds’ antitumour activity. Collectively, our findings suggest that these polyfluoroalkyl phosphonates represent promising ROS-modulating chemotherapeutic candidates with unique mechanisms of action that may complement existing GBM treatment strategies.

**Supplementary Information:**

The online version contains supplementary material available at 10.1038/s41598-025-22754-0.

## Introduction

Glioblastoma (GBM, IDH-wildtype diffuse astrocytoma, WHO grade IV) is the most prevalent primary tumour of the central nervous system (CNS), comprising 14.5% of all and 48.6% of malignant CNS tumours^1^. GBM remains the most aggressive primary malignant brain tumour in adults with a median survival time in registry databases of 6–10 months and 14.6–21.1 months when anticancer treatment is included^2^. Only 3% to 5% of patients survive more than three years, and reports of survival exceeding five years are sporadic^3^. Despite the efforts made by clinicians and researchers, no major clinical advances have been achieved in increasing overall patient survival since establishing Stupp protocol 20 years ago (12.1 vs. 14.6 months)^4,5^. This standard of care includes maximal safe resection and radiotherapy with concurrent and adjuvant chemotherapy with temozolomide (TMZ). Despite multimodal treatments, recurrence is almost inevitable, partially due to the difficulty of complete surgical removal, with a median interval of less than 10 months^6–8^. The treatment options are limited at recurrence, with the unavailability of a universal standard of care for recurrent GBM. Therefore, re-resection, re-irradiation and systemic chemotherapy with TMZ rechallenge, nitrosoureas, bevacizumab, and tumour treating fields (TTF) or clinical trial enrolment to test experimental drugs are considered for all recurrent patients.

TMZ remains the only available first-line chemotherapeutic agent for GBM. As one of the few chemotherapeutic drugs capable of crossing the blood–brain barrier (BBB), with approximately 20% of the injected dose reaching the brain, TMZ offers a modest survival benefit by increasing patient lifespan by only about 2.5 months^4^. TMZ chemotherapy and radiotherapy share common pathways to GBM cell death, inducing DNA damage either directly or indirectly by generating reactive oxygen species (ROS). Although the cytotoxic effects of TMZ is mainly attributed to the induction of DNA damage, *O*^6^-methylguanine, oxidative stress induced by TMZ was demonstrated to play a decisive role in its cytotoxic effects^9–11^. Inhibition of reactive oxygen species (ROS) prevented cytotoxic effects of TMZ^9,11^. Moreover, TMZ-sensitive and resistant GBM cells display differences in antioxidant system with the resistant cells displaying upregulation of the antioxidant system indicating key role of ROS detoxification in the TMZ resistance mechanism^9–11^.

ROS play a dual role in GBM, exerting both tumour-promoting and tumour-suppressing effects. This paradox has spurred the development of ROS-inducing agents designed to elevate ROS levels beyond the threshold of cellular tolerance, thereby selectively targeting GBM cells^10^. Small molecule compounds that modulate redox homeostasis have been investigated in both preclinical and early-phase clinical settings^10,12–14^. In preclinical models these compounds have individually induced GBM cell death or enhanced GBM cell sensitivity to the conventional therapies^10^. Early-phase clinical trials have begun to assess their efficacy, though no ROS-modulating small molecule has yet significantly improved the standard of care^15^. Nonetheless, small molecules remain the predominant drug class under clinical evaluation, and the rational design of novel compounds, particularly in combination therapies, holds promise for improving GBM treatment outcomes.

The focus on small molecule compounds in the design of GBM chemotherapeutic candidates are highly desired due to the presence of the BBB, which allows only certain drugs (i.a. with molecular mass below 400–500 Da) to enter the brain^16^. This restricts the clinical application of most anticancer drugs for treating brain tumours. Therefore, the development of new small molecule compounds that can modulate the redox status of GBM cells and induce cell death through oxidative stress is urgently needed.

**Fig. 1:**
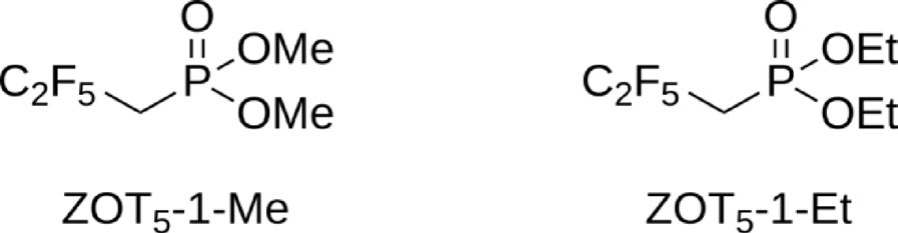
Chemical structure of ZOT_5_-1-Me and ZOT_5_-1-Et.

Simple dialkyl 2,2,3,3,3-pentafluoropropylphosphonates (referred to as ZOTs, Fig. 1) were designed in our laboratory as small molecular weight anticancer drug candidates with the potential to permeate the BBB. We hypothesized that an optimal scaffold for GBM therapy should combine: (a) low molecular weight; (b) lipophilic fragments to facilitate BBB permeation; (c) a brain-targeting element; and (d) an innovative lead structure distinct from existing drugs. Polyfluoroalkylated motifs, known to increase lipophilicity and BBB passage, were therefore incorporated^17^. Because poly/perfluoroalkyl substances are broadly membrane-permeant rather than BBB-specific, these fragments were coupled to a phosphonate group to exploit the anionic chemical delivery system for brain targeting and retention^18^. Initial cytotoxicity screening revealed that two ZOTs, specifically ZOT_5_-1-Me and ZOT_5_-1-Et, exhibit high potency against GBM cells^19^. ZOTs represent a new class of small molecular weight compounds that combine polyfluoroalkyl and phosphonic moieties. Due to their unique chemical structure and simplicity, predicting the molecular mechanism behind their cytotoxic properties is challenging, as no studies have been conducted on similarly structured compounds. The promising cytotoxicity results of ZOT_5_-1-Me and ZOT_5_-1-Et against GBM cells, along with computational study, encouraged us to further explore the molecular mechanism underlying their cytotoxic effects in GBM^19^.

## Materials and methods

### Cell line

Human GBM cell line, U-87 MG was purchased from ECACC (Sigma-Aldrich Chemie GmbH, Munich, Germany) and was maintained in Eagle’s Minimum Essential Medium (EMEM) medium supplemented with 10% inactivated fetal bovine serum (FBS) (Biowest, Nuaillé, France), 2 mM L-glutamine (Merck Life Science, Darmstadt, Germany), 1% non-essential amino acids (NEAA, Merck Life Science), 1 mM sodium pyruvate (NaP, Merck Life Science), and 100 units/mL penicillin (Merck Life Science) and 100 μg/mL streptomycin (Merck Life Science). Cells were incubated in a humidified atmosphere of 5% CO_2_ at 37 °C and sub-cultured once to twice per week to maintain exponential growth. The cultured U-87 MG cells from passages 3–20 were used for experiments.

### Compounds

ZOT_5_-1-Me and ZOT_5_-1-Et were synthesized according to the general procedures published elsewhere^19^ with minor modifications. The intermediate hydroxyphosphonates were additionally purified by distillation. The final distilled ZOT_5_-1-Me and ZOT_5_-1-Et were purified by column chromatography using sequential elution with n-heptane, dichloromethane and acetonitrile. The NMR spectra were consistent with the literature. For biological assays ZOT_5_-1-Me and ZOT_5_-1-Et were freshly dissolved in DMSO to a stock concentration of 100 mM. Subsequent dilutions were conducted in cell culture medium.

### Compound screening

Synthesized compounds ZOT_5_-1-Me and ZOT_5_-1-Et were submitted to National Cancer Institute (NCI, Bethesda, Maryland, U.S.A.) under the Developmental Therapeutic Program (DTP). Compounds were screened in a panel of 59 cancer cell lines, including glioma cell lines (SF-268, SF-295, SF-539, SNB-19, SNB-75, and U251) at a concentration of 10 µM in the one-dose screen and concentrations 0.01, 0.1, 1, 10 and 100 µM in the five-dose screen according to the procedure (https://dtp.cancer.gov/discovery_development/nci-60/methodology_HTS384.htm). The sulforhodamine B assay was used for the determination of cell density relative to the no-drug control and relative to the number of cells at time zero. The results were utilized to generate dose–response curves, plotting the logarithm (base 10) of the compound concentration against the percentage of growth inhibition. For each cell line, three parameters were determined: GI50, TGI, and LC50. The GI50 (growth inhibitory activity) indicates the concentration at which cell growth is reduced by 50%. The TGI (total growth inhibition) represents the concentration required to completely halt cell proliferation. The LC50 (cytotoxic activity) reflects the compound concentration that leads to a 50% reduction in the initial cell population after 48 h of exposure.

### Cell treatment

U-87 MG cells were seeded onto tissue culture plates or dishes at a density of 5 × 10^4^/mL in EMEM medium and allowed to attach for 24 h. After overnight growth, cells were treated with 0.03125**%** DMSO (as a vehicle) and ZOTs in a concentration range 0.98–250 µM for 4–48 h as indicated beneath. Negative control cells were incubated in medium only.

### Cell viability assay

Logarithmically growing cells (5 × 10^3^) were seeded into 96-well plates and treated with ZOTs in the concentration range 0.98–250 µM for 4–48 h. After the desired time elapsed, 10 μL of Cell Counting Kit-8 (CCK-8, Merck Life Science) was added to each well to obtain a final concentration of 0.5 mg/mL CCK-8 and plates again were incubated in 5% CO_2_ at 37 °C for 2 h. Next, absorbance was measured at 450 nm with a microplate reader Synergy HT (Bio-Tek Instruments, Winooski, VT, USA).

### Annexin V and propidium iodide apoptosis assay

Double staining of cells with Annexin V-FITC and Propidium Iodide (PI) was used to assay apoptosis. This method is a useful tool for distinguishing viable cells (Annexin V^-^/PI^-^), early apoptotic cells (Annexin V^+^/PI^-^), late apoptotic cells (Annexin V^+^/PI^+^), and necrotic cells (Annexin V^-^/PI^+^). Visualization of cells stained with Annexin V-FITC and PI was applied according to the manufacturer’s protocol (FITC Annexin V Apoptosis Detection Kit I, Becton Dickinson, Franklin Lakes, NJ, USA). Briefly, 1.5 × 10^5^ cells were seeded onto 6-well plates and allowed to attach. Next, the cells were treated with the indicated concentrations of ZOTs for 4, 24 and 48 h. After treatment, the cells were washed twice with cold PBS and resuspended in 300 μL binding buffer containing 3 μL of Annexin V-Alexa Fluor™ 488 at 27 µg/mL and 3 μL of PI at 50 μg/mL and stained for 15 min at room temperature in the dark. Finally, at least 10^4^ cells were analysed using a BD FACSymphony™ A1 Cell Analyzer (Becton Dickinson, Franklin Lakes, NJ, USA) and BD FACSDiva™ Software v9.0.2. FITC conjugated antibody was excited with the 488 nm laser and emission detected with a 530/30 bandpass filter. PI was excited with the 561 nm laser and its emission detected with a 610/20 bandpass filter. A dual parameter dot plot of forward (FSC-A) and side scatter peak areas (SSC-A) were used to identify intact cells from debris. Forward scatter peak area and height (FSC-A and FSC-H) and side scatter peak area and height (SSC-A and SSC-H) were used to identify single cells and exclude doublets. Data were analysed in FlowJo v7.6.4 software (FlowJo LLC, Ashland, OR, USA). The positive control cells were incubated with 20 μM camptothecin (CPT) for 48 h at 37 °C.

### Caspase activity assay

U-87 MG cells were seeded on a 96-well black plate in a count of 3.750 per well in 75 µL medium. Then the cells were incubated with ZOTs for 4, 24 and 48 h at 37 °C in 5% CO_2_. Following the treatments the cells were subjected to caspase 3/7, 8, and 9 activities measurement with Caspase-Glo assay kits (Promega, Madison, WI, USA) according to the manufacturer’s protocol. Briefly, the plates containing cells were removed from the incubator and allowed to equilibrate to room temperature for 30 min. One volume of Caspase-Glo reagent was added to each well, the content of well was gently mixed with a plate shaker at 400 rpm for 30 s. The plate was then incubated at room temperature for 1 h. The luminescence of each sample was measured with a microplate reader Synergy HT (Bio-Tek Instruments, USA). The positive control cells were incubated with 20 μM camptothecin (CPT) at 37 °C. Caspase activities were expressed as a percentage compared to that of control cells (100%).

### Caspase inhibition

The inhibition of caspases was conducted by a cell-permeant pan-caspase inhibitor Z-VAD-FMK (Promega, Madison, WI, USA) that irreversibly binds to the catalytic site of caspase proteases. Z-VAD-FMK was added to the culture medium at a concentration of 10 μM, according to the manufacturer’s recommendations for 48 h. Controls were generated using DMSO as an inhibitor vehicle at the same concentration. The effectiveness of the caspase inhibitor was verified with Caspase-Glo assays.

### Intracellular ROS assay

The analysis of total intracellular ROS was conducted using 5-(and-6)-chloromethyl-2′,7′-dichlorodihydrofluorescein diacetate, acetyl ester (CM-H_2_DCFDA) (Invitrogen, Waltham, MA, USA) as described previously^20^. Briefly, the U-87 MG cells were detached with trypsin on the day of the analysis. Cells in suspension (5 × 10^5^ cells per 1 mL medium) were washed twice with HBSS (Biowest, Nuaillé, France) and stained with 9 µM CM-H_2_DCFDA in HBSS for 30 min at 37 °C in the dark. Then, the cells were washed twice with HBSS and treated with an increasing concentration of ZOTs for 2 h at 37 °C in the dark. Hydrogen peroxide (H_2_O_2_) at a concentration of 500 µM was used as positive control. Pre-incubation with 1 mM N-acetyl-L-cysteine (NAC) for 1 h was used to scavenge ROS. Then, the fluorescence intensity of 2’,7'-dichlorofluorescein (DCF) was measured with the excitation and emission set at 485/20 nm and 528/20 nm, respectively, using a Synergy HT spectrophotometer (BioTek Instruments, Winooski, VT, USA).

### Mitochondrial membrane potential (ΔΨm)

The change in the ΔΨm was detected by staining with 5,5ʹ,6,6ʹ-tetrachloro-1,1ʹ,3,3ʹ-tetraethylbenzimidazolylcarbocyanine iodide (JC-1) (Sigma-Aldrich Chemie GmbH, Munich, Germany) as described elsewhere^21^. Briefly, 0.86 × 10^6^ cells were seeded onto 100 mm cell culture dishes and allowed to attach. Next, the cells were treated with the indicated concentrations of ZOTs for 48 h at 37 °C. The positive control cells were incubated with 25 μM carbonyl cyanide 3-chlorophenylhydrazone (CCCP), a mitochondrial uncoupling agent, for 48 h at 37 °C. After the treatment, the cells were washed with PBS and detached with trypsin on the day of the analysis. Cells in suspension (1 × 10^6^ cells per 1 mL) were washed twice with HBSS and stained with 5 µM JC-1 in HBSS (Biowest, Nuaillé, France) for 30 min at 37 °C in the dark. After this incubation period, cells were washed twice with HBSS and 10^5^ cells were transferred to a 96-well black plate for fluorescence measurement. The fluorescence intensity was measured with Synergy HT spectrophotometer (BioTek Instruments, Winooski, VT, USA) with the following settings: excitation at 530/25 nm and emission at 590/35 nm for JC-1 dimers and excitation at 485/20 nm and emission at 528/20 nm for JC-1 monomers. The value of ΔΨm was expressed as the ratio of JC-1 dimer fluorescence to monomer fluorescence (measurements at 590 nm/528 nm). The JC-1 stained cells were imaged immediately after the dye was washed with HBSS with an inverted Olympus IX70 fluorescence microscope (Olympus, Tokyo, Japan).

### Comet assay

On the day of the experiment, U-87 MG cells were harvested using trypsin and subsequently resuspended at a concentration of 5 × 10^4^ cells per mL of culture medium. The cells were then exposed to a gradient of ZOT compound concentrations for 2 h. DNA damage was assessed using the comet assay under three different pH conditions: neutral (pH 9), pH 12.1, and alkaline (pH > 13), following previously published protocols^20^. The neutral version of the assay is selective for detecting DNA double-strand breaks (DSBs), while the pH 12.1 condition allows visualization of both single- and double-strand breaks. In contrast, the alkaline version identifies a broader spectrum of lesions, including alkali-labile sites and DNA strand breaks.

Following treatment, cells were mixed with 0.75% low melting point (LMP) agarose at 37 °C and rapidly layered onto microscope slides precoated with 0.5% normal melting point (NMP) agarose. A cover slip was applied to evenly distribute the cell-agarose suspension. The slides were then placed on ice to allow the agarose to solidify. Once solidified, the coverslips were carefully removed, and the slides were immersed for 1 h in lysis buffer containing 2.5 M NaCl, 100 mM EDTA, 1% Triton X-100, and 10 mM Tris at pH 10 to remove membranes and proteins.

For the alkaline version of the assay, slides were transferred into ice-cold buffer consisting of 300 mM NaOH and 1 mM EDTA (pH > 13) for 20 min to unwind the DNA. Electrophoresis was then conducted in the ice-cold buffer of 30 mM NaOH, 1 mM EDTA for 20 min under an electric field of 0.73 V/cm (32 mA).

In the pH 12.1 variant, the denaturation step was similarly performed for 20 min in a buffer made of 300 mM NaOH and 1 mM EDTA, with the pH adjusted to 12.1 using glacial acetic acid. Electrophoresis followed under the same conditions: 20 min at 4 °C and 0.73 V/cm.

For the neutral conditions, the slides were equilibrated for 20 min in an ice-cold buffer containing 100 mM Tris and 300 mM sodium acetate, with pH adjusted to 9.0 using glacial acetic acid. This was followed by electrophoresis for 60 min at 4 °C, using a field of 0.41 V/cm (100 mA).

Following electrophoresis, the slides were rinsed with distilled water, air-dried, stained with DAPI at a concentration of 4 µg/mL, and covered with cover slips. Fluorescent imaging was conducted at 200 × magnification using an Eclipse fluorescence microscope (Nikon, Tokyo, Japan) equipped with a ProgRes MF cool monochrome camera (JENOPTIK, Jena, Germany) and connected to a Lucia Comet Assay 7.30 image analysis system (Laboratory Imaging, Prague, Czech Republic). For each sample, 50 comets were randomly selected for analysis, and the percentage of DNA in the tail region was quantified as an indicator of DNA damage, including strand breaks and alkali-labile sites^22^.

### DNA repair

To examine DNA repair, the cells were prepared as for comet assay and after the treatment with ZOTs, cells were washed and resuspended in fresh medium containing 10% FBS. Cells were allowed to repair for 5, 10, 15, 30, 60, 90, and 120 min and the alkaline version of comet assay was performed. The kinetics of DNA repair was quantified by determination of the extent of residual DNA damage at each time-point.

### Plasmid relaxation assay

The plasmid relaxation assay was performed according to established methodology^23^. Plasmid pUC19 was extracted from *E.coli* DH5α using the Isolate II Plasmid Mini Kit (Meridian Bioscience, Cincinnati, OH, USA) in accordance with the manufacturer’s protocol. The concentration and purity of the obtained plasmid DNA were assessed using the spectrophotometer and verified by agarose gel electrophoresis. In its native state, pUC19 is predominantly in a supercoiled (covalently closed circular, CCC) conformation, which exhibits elevated electrophoretic mobility. To generate the linear (L) form, the plasmid was digested with the *Pst*I restriction enzyme (New England Biolabs, Ipswich, MA, USA) according to the manufacturer’s protocol. Changes in topological structure between the CCC and L forms results in distinct migration patterns during electrophoresis. To evaluate the capacity of ZOT compounds to cause DNA strand breaks, 250 ng of plasmid DNA was incubated with increasing concentrations of the compounds at 37 °C for 24 h. Post-incubation, the samples were immediately subjected to electrophoresis on 0.8% agarose gels followed by ethidium bromide staining. DNA bands were visualized under UV illumination at 302 nm, captured using a CCD imaging system, and analysed using GeneTools software (Syngene, Cambridge, UK). A 1 kb DNA ladder (GeneRuler 1 kb DNA Ladder, Thermo Scientific, Waltham, MA, USA) served as a molecular weight marker.

### γH2AX analysis using flow cytometry

The analysis of γH2AX with flow cytometry was conducted as described previously^24^. On the day of the treatment, U-87 MG cells were detached with trypsin. Cells in suspension (3 × 10^5^ cells in 5 mL medium) were treated with increasing concentrations of ZOTs for 2 h. After treatment, cells were washed with PBS, fixed and permeabilized with freshly prepared 1× Cytofix/Cytoperm Buffer (BD Biosciences, San Diego, CA, USA) for 40 min at 4 °C. Then, cells were washed twice with 1× Perm/Wash Buffer (BD Biosciences) and incubated with rabbit monoclonal anti-phospho-H2AX (Ser139) antibody (#9718, Cell Signaling Technology, Danvers, MA, USA) diluted 1:250 in 1× Perm/Wash Buffer (BD Biosciences) for 30 min at 37 °C. Next, cells were washed twice with 1× Perm/Wash Buffer (BD Biosciences) and incubated with secondary goat anti-rabbit IgG (H + L) Alexa Fluor 488-conjugated antibody (#A-11070, Invitrogen, Waltham, MA, USA) diluted 1:400 in 1× Perm/Wash Buffer (BD Biosciences) for 30 min at 37 °C in the dark. Cells were washed again twice with 1× Perm/Wash Buffer (BD Biosciences), re-suspended in PBS and subjected to flow cytometric analysis at an excitation wavelength of (488 nm) employing BD FACSymphony™ A1 Cell Analyzer (Becton Dickinson, San Jose, CA, USA) equipped with BD FACSDiva™ Software v9.0.2. FITC conjugated antibody was excited with the 488 nm laser and emission was detected with a 530/30 bandpass filter. A dual parameter dot plot of forward (FSC-A) and side scatter peak areas (SSC-A) were used to identify intact cells from debris. Forward scatter peak area and height (FSC-A and FSC-H) followed by side scatter peak area and height (SSC-A and SSC-H) were used to identify single cells and exclude doublets. Data were analysed in FlowJo v7.6.4 software (FlowJo LLC, Ashland, OR, USA). The positive control cells were incubated with 50 μM etoposide (Etop) for 4 h at 37 °C.

### Western blot

Western blot was conducted as described previously^25^. On the day of the treatment, U-87 MG cells were detached with trypsin. Cells in suspension (2 × 10^5^ cells per 1 mL medium) were treated with increasing concentrations of ZOTs for 2 h and the Western blot for the assessment of γH2AX level was performed.

For the analysis of cell cycle proteins, 0.86 × 10^6^ cells were seeded onto 100 mm cell culture dishes and allowed to attach. Next, the cells were treated with the 7.8 µM ZOT_5_-1-Me and 15.6 µM ZOT_5_-1-Et for 24 or 48 h at 37 °C. After the treatment, the cells were washed with PBS and detached with trypsin.

Following the treatments, cells were washed with ice cold PBS and lysed in ice cold RIPA buffer (50 mM Tris HCl pH 8, 150 mM NaCl, 0.5% sodium deoxycholate, 1% Nonidet P-40, 0.1% SDS, 1 mM EDTA) containing 1 mM phenylmethanesulfonyl fluoride (PMSF) and 1× protease and phosphatase inhibitor cocktail (Sigma-Aldrich Chemie GmbH, Munich, Germany). The protein concentration in cell lysates was determined using the Bradford method^26^. The 80 µg (for γH2AX) or 40 µg (for p53 and p21) of proteins was separated on 10–12% SDS-PAGE and transferred to PVDF transfer membrane (ThermoFisher Scientific, Waltham, MA, USA). The membranes were blocked with 5% milk in 0.1% Tween-20/TBS (Tris-buffered saline) for 1 h at room temperature and they were incubated with rabbit monoclonal anti-phospho-H2AX (Ser139) antibody (#9718, Cell Signaling Technology, Danvers, MA, USA) diluted 1:1000 in 5% milk and 5% BSA in 0.1% TBST or anti-phospho-p53 (Ser15) (#9284, CST) diluted 1:1000 in 5% milk in 0.1% TBST, or anti-p21 (#2947, CST) or anti-p53 (#2527, CST) diluted 1:1000 in 5% BSA in 0.1% TBST overnight at 4 °C. Goat anti-rabbit antibody conjugated with horseradish peroxidase (#7074, CST) were used as secondary antibodies. For loading, the control detection of β-actin was performed with anti-β-actin antibody (sc-477778, SantaCruz Biotechnology, Santa Cruz, CA, USA) diluted 1:4000 in 5% milk in 0.1% TBST. The bands were visualized with Clarity Western ECL Substrate (Bio-Rad, Hercules, CA, USA) and Gel-Pro Analyzer (Microchem Laboratory, Round Rock, TX, USA). The band quantification was performed using ImageJ software v1.54 g (National Institutes of Health, Bethesda, MD, USA) and the band intensities were normalized against β-actin.

### Cell proliferation assay

The analysis of proliferation rate was conducted as described elsewhere^27^. Briefly, 5 × 10^4^ cells were seeded onto 6-well plates and allowed to attach. The number of cells on the day of seeding was considered as the number of cells at the starting time point (time 0). Next, cells were treated with the indicated concentrations of ZOTs for 24, 48, 72, and 96 h. Following the treatment, cells were detached with trypsin and the number of viable and non-viable cells was manually counted in a hemocytometer using trypan blue exclusion method. Proliferation curves were generated by plotting viable cell counts against the time of culture. Population doubling times (PDTs) were determined based on the cell proliferation assay results and calculated using the doubling time online calculator^28^.

### Cell cycle analysis

The analysis of cell cycle was conducted as described previously^20^. Briefly, 1.5 × 10^5^ cells were seeded onto 6-well plates and allowed to attach. Next, the cells were treated with the indicated concentrations of ZOTs for 24 and 48 h. After treatment, the cells were collected, washed twice with PBS, resuspended in PBS to a final concentration of 10^6^ cells/mL and allowed to cool on ice for 15 min. One volume of − 20 °C absolute ethanol was added to each sample and the samples were stored at 4 °C until analysis. At that time, the cells were pelleted (400 × *g*, 20 min) and resuspended in a staining solution containing 50 µg/mL PI (81845, Sigma-Aldrich Chemie GmbH, Munich, Germany) and 50 U/mL RNase A (EURx, Gdansk, Poland) in PBS. Samples were incubated at 37 °C for at least 30 min in the dark prior to analysis by fluorescence-activated cell sorting (FACS) performed on the BD FACSymphony™ A1 Cell Analyzer (Becton Dickinson, San Jose, CA, USA) and BD FACSDiva™ Software v9.0.2. PI was excited with the 561 nm laser and its emission detected with a 610/20 bandpass filter. A dual parameter dot plot of forward (FSC-A) and side scatter peak areas (SSC-A) were used to identify intact cells from debris. Forward scatter peak area and height (FSC-A and FSC-H), side scatter peak area and height (SSC-A and SSC-H), and PI peak area and half wide (PE-A and PE-W) were used to identify single cells and exclude doublets. Data were analysed in FlowJo v7.6.4 software (FlowJo LLC, Ashland, OR, USA). The positive control cells were incubated with 200 ng/mL nocodazole (NOC) for 18 h at 37 °C.

### Data analysis

Statistical analyses were conducted using GraphPad Prism 8.0.1 Software (GraphPad Software, San Diego, CA, USA). For datasets with sample sizes *n* < 30, comparisons were conducted using the Mann–Whitney U test. For sample sizes *n* > 30, an unpaired Student’s *t*-test was applied, provided that data distribution satisfied the Shapiro–Wilk normality criterion. Results are reported as mean ± standard error of the mean (SEM). Unless otherwise specified, tests were two-tailed, and a statistical significance was assigned with *p-value ≤ 0.05, **p-value ≤ 0.01, ***p-value ≤ 0.001, *ns*, not significant.

### Data availability

The data generated in this study are available at Zenodo (10.5281/zenodo.16272119). Drug response profiles obtained in this study are available at NCI/DTP repository (https://dtp.cancer.gov/dtpstandard/dwindex/index.jsp); at NSC 835966 (ZOT_5_-1-Me; https://dtp.cancer.gov/dtpstandard/servlet/dwindex?searchtype=NSC&searchlist=835966) and NSC 835967 (ZOT_5_-1-Et, https://dtp.cancer.gov/dtpstandard/servlet/dwindex?searchtype=NSC&searchlist=835967).

## Results

### ZOT_5_-1-Me and ZOT_5_-1-Et are cytostatic and cytotoxic for glioblastoma and astrocytoma cells

**Fig. 2:**
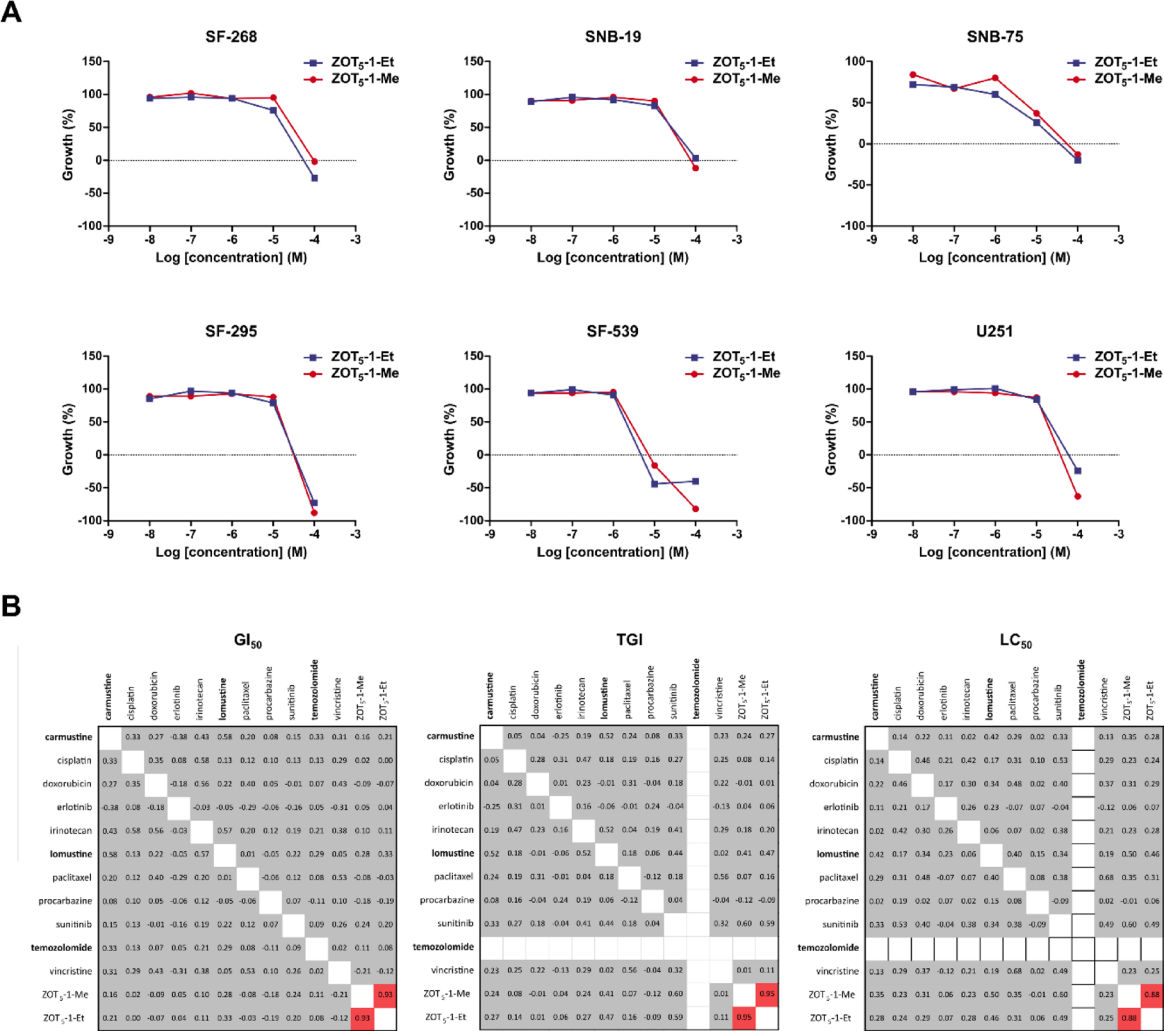
ZOT_5_-1-Me and ZOT_5_-1-Et induced growth inhibition and lethality in glioblastoma and astrocytoma cell lines and COMPARE analysis of their mechanisms of action relative to reference anticancer drugs. (**A**) Cells were treated with an increasing concentration (10^–8^ to 10^–4^ M) of ZOT_5_-1-Me and ZOT_5_-1-Et for 48 h, and the analysis was performed thanks to the NCI-60 Cell Screen. Five dose–response curves in 3 glioblastoma (SF-295, SF-539, and SNB-75) and 3 astrocytoma (SF-268, SNB-19, and U251) cell lines are shown. NCI-60 analysis allows for the detection of both growth inhibition (values between 0 and 100) and lethality (values less than 0). A value of 100% represents no growth inhibition. The value of 0% indicates no cell growth throughout the experiment, corresponding to the number of cells at the beginning. The value of −50% means 50% lethality, and the value of 100% means that all cells are dead by treatment. GI_50_, the concentration required for 50% cell growth inhibition; TGI, the concentration required for total inhibition (0%); LC_50_, the concentration required for 50% cell death. (**B**) Comparison of the mechanism of ZOT_5_-1-Me and ZOT_5_-1-Et to FDA-approved and non-FDA-approved GBM anticancer drugs by COMPARE plots. ZOT_5_-1-Me and ZOT_5_-1-Et have a unique mechanism compared to known chemotherapeutics. High PCC (Pearson correlation coefficient) (> 0.8) shown in red in the matrix COMPARE figure, indicates these two drugs have similar mechanisms of anticancer action.

The tumour growth inhibition properties of ZOT_5_-1-Me and ZOT_5_-1-Et were evaluated on human cancer cell lines at the NCI as part of the Developmental Therapeutics Program (DTP). Both compounds met the NCI-60 one-dose (10 μM) screening criteria allowing them to advance to the five-dose testing stage. Table 1 and Fig. 2A present the five-dose assay results for the anticancer activity of these compounds against glioma cell lines. Results presented are expressed as the 50% growth inhibition concentration (GI_50_), 50% lethal concentration (LC_50_) and total growth inhibition (TGI), compared to the values obtained for untreated control cells (Table 1). The GI_50_ results indicated that both ZOT_5_-1-Me and ZOT_5_-1-Et exhibited antiproliferative effects against all tested GBM (SF-295, SF-539, and SNB-75) and astrocytoma (SF-268, SNB-19, and U251) cell lines. ZOT_5_-1-Me achieved total growth inhibition across all tested cell lines, while ZOT_5_-1-Et inhibited 100% of growth in all the cell lines except astrocytoma SNB-19 cell line. The LC_50_ data showed that ZOT_5_-1-Me was cytotoxic for the SF-295, SF-539, and U251 cell lines, and ZOT_5_-1-Et demonstrated a significant lethal effect on the GBM SF-295 cell line.

**Table 1:** The 50% growth inhibition (GI_50_), total growth inhibition (TGI) and 50% lethal (LC_50_) concentrations of ZOT_5_-1-Me, ZOT_5_-1-Et, carmustine (BCNU), lomustine (CCNU), and temozolomide (TMZ) towards the glioblastoma and astrocytoma cell lines. Results were obtained in the NCI-60 five-dose screen.

Indices	SF-268	SF-295	SF-539	SNB-19	SNB-75	U251
**ZOT**_**5**_**-1-Me**						
GI_50_	24.2 µM	16.5 µM	2.55 µM	24.8 µM	4.93 µM	17.6 µM
TGI	64.2 µM	31.8 µM	7.21 µM	77.0 µM	55.3 µM	38.0 µM
LC_50_	> 100 µM	61.0 µM	32.7 µM	> 100 µM	> 100 µM	82.1 µM
**ZOT**_**5**_**-1-Et**						
GI_50_	17.9 µM	15.6 µM	2.02 µM	26.2 µM	1.95 µM	20.7 µM
LC_50_	54.4 µM	33.2 µM	4.75 µM	> 100 µM	36.7 µM	60.2 µM
TGI	> 100 µM	70.9 µM	> 100 µM	> 100 µM	> 100 µM	> 100 µM
**BCNU**						
GI_50_	13.7 µM	4.6 µM	15.4 µM	17.5 µM	27.0 µM	12.0 µM
TGI	212.8 µM	422.7 µM	170.6 µM	218.3 µM	140.9 µM	240.4 µM
LC_50_	350.8 µM	473.2 µM	239.9 µM	305.5	342.0 µM	346.7 µM
**CCNU**						
GI_50_	24.6 µM	32.0 µM	23.9 µM	52.5 µM	37.2 µM	31.6 µM
TGI	124.2 µM	117.5 µM	85.1 µM	121.9 µM	113.8 µM	104.2 µM
LC_50_	373.3 µM	266.1 µM	219.8 µM	257.0 µM	267.9 µM	258.8 µM
**TMZ**						
GI_50_	> 100 µM	> 100 µM	> 100 µM	> 100 µM	70.8 µM	> 100 µM
TGI	> 100 µM	> 100 µM	> 100 µM	> 100 µM	> 100 µM	> 100 µM
LC_50_	> 100 µM	> 100 µM	> 100 µM	> 100 µM	> 100 µM	> 100 µM

To compare the efficacy of these compounds with existing therapies, GI_50_, TGI, and LC_50_ values for three FDA-approved GBM drugs (TMZ, lomustine [CCNU], and carmustine [BCNU]) were retrieved from the DTP database and compared to the values for ZOT_5_-1-Me and ZOT_5_-1-Et (Table 1). The TGI values revealed that both ZOT_5_-1-Me and ZOT_5_-1-Et showed higher efficacy than the FDA-approved GBM drugs across all cell lines, except for the ZOT_5_-1-Et in SNB-19 cell line (data not available). Overall, both ZOT_5_-1-Me and ZOT_5_-1-Et demonstrated greater efficacy than most FDA-approved drugs against GBM.

Additionally, the COMPARE algorithm was used to evaluate the similarity of the biological response pattern of ZOT_5_-1-Me and ZOT_5_-1-Et with both FDA-approved drugs and chemotherapeutics undergoing combination clinical trials for GBM (Fig. 2B). The algorithm was used to assess similarities in anticancer mechanisms, quantified as the Pearson correlation coefficient (PCC). For both compounds, nearly all PCC values were below or equal to 0.6 for GI_50_, TGI, and LC_50_ values, indicating minimal correlation with the mechanisms of known chemotherapeutics. These findings suggest that the mechanisms of action of ZOT_5_-1-Me and ZOT_5_-1-Et are unique and distinct from those of established chemotherapeutic agents. Consequently, further investigations were undertaken to explore the novel anticancer mechanisms of ZOT_5_-1-Me and ZOT_5_-1-Et.

### ZOT_5_-1-Me and ZOT_5_-1-Et induced caspase-dependent and -independent cell death in U-87 MG cells

**Fig. 3:**
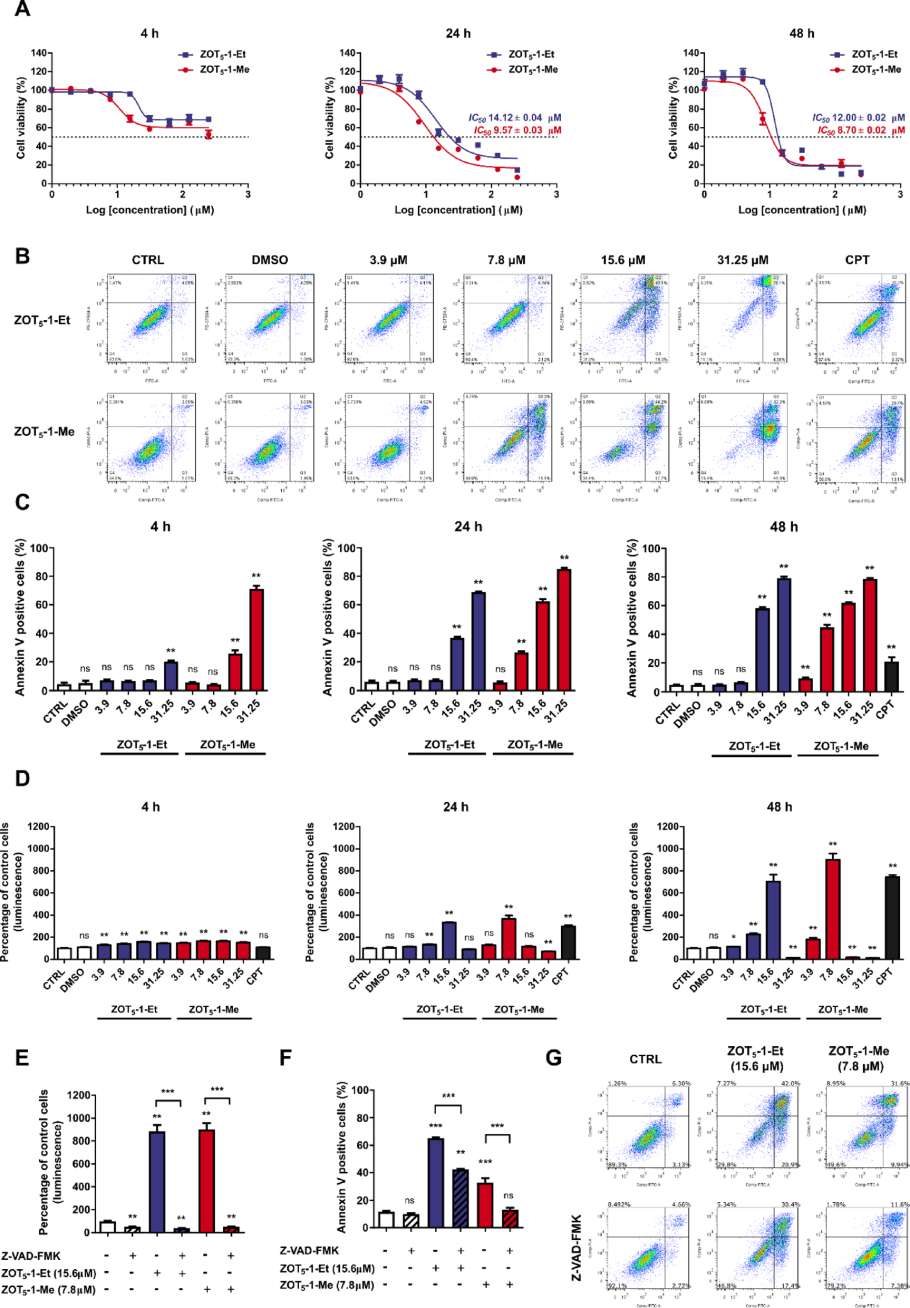
ZOT_5_-1-Me and ZOT_5_-1-Et induced cell death and apoptosis in U-87 MG cells. (**A**) The decreased viability of U-87 MG cells after treatment with an increasing concentration (0.95–250 µM) of ZOT_5_-1-Me and ZOT_5_-1-Et for 4–48 h was evaluated by CCK-8 assay. Cell viability curves and corresponding IC_50_ values are visible. The dashed line represents a 50% reduction in CCK-8 signal. The data were normalized to control cells (CTRL, 100%, *n* = 9). (**B,C**) ZOT_5_-1-Me and ZOT_5_-1-Et induced externalisation of phosphatidylserine in U-87 MG cells as evaluated by Annexin V/PI assay followed by flow cytometry (FACS) quantification (*n* = 6). Camptothecin (CPT, 20 µM) was used as positive control. (**B**) Representative FACS dot plots after 48 h treatment with ZOTs are presented with the indicated percentages of necrotic (Q1), late-apoptotic (Q2), early-apoptotic (Q3), and viable cells (Q4). Apoptosis was presented as a percentage of Annexin V-positive cells (Q2 + Q3). FACS dot plots after 4 h and 24 h treatments are presented in Supplementary material (**D**) ZOT_5_-1-Me and ZOT_5_-1-Et activated caspase 3/7 in U-87 MG cells as estimated by Caspase-Glo 3/7 assay (*n* = 6). The data were normalized to control cells (CTRL, 100%, *n* = 6). CPT (20 µM) was used as positive control. (**E**–**F)** Pre-incubation with pan-caspase inhibitor Z-VAD-FMK (10 µM, 48 h) decreased ZOT_5_-1-Me and ZOT_5_-1-Et-evoked apoptosis in U-87 MG cells as determined by Caspase-Glo 3/7 assay (**E**) and Annexin V/PI assay (**F**) after 48 h treatment with ZOTs (*n* = 6). (**G**) Representative FACS dot plots are presented. DMSO was used as a solvent control. Results presented as bar plots with mean ± SEM; ^*^p < 0.05; ^**^p < 0.01; ^***^p < 0.001, *ns* – not statistically significant.

Based on our previous findings^19^, U-87 MG was identified as the most sensitive cell line to both ZOT_5_-1-Me and ZOT_5_-1-Et, prompting its selection for further investigation. To assess the cytotoxic effects of these compounds, U-87 MG cells were exposed to increasing concentrations of ZOT_5_-1-Me and ZOT_5_-1-Et (0–250 μM) for 4 h, 24 h, and 48 h, followed by cell viability assessment using CCK-8 assay. Significant cytotoxicity was detected as early as 4 h post-treatment with both compounds (Fig. 3A). After 24 h of treatment, dose-dependent cytotoxic effects were evident, with IC_50_ values of 9.57 μM for ZOT_5_-1-Me and 14.12 μM for ZOT_5_-1-Et, indicating higher potency of ZOT_5_-1-Me. Extending the treatment to 48 h led to IC_50_ values of 8.70 μM for ZOT_5_-1-Me and 12.00 μM for ZOT_5_-1-Et, reflecting a marginal increase in cytotoxicity compared to the 24-h exposure. These findings suggest that ZOTs effectively exert their cytotoxic action within relatively short incubation periods, and extending treatment beyond 48 h may not provide significant additional benefits.

To investigate whether the observed cytotoxicity was associated with apoptosis induction, an Annexin V-FITC/PI assay was performed following the exposure of U-87 MG cells to ZOT_5_-1-Me and ZOT_5_-1-Et for 4 h, 24 h, and 48 h. Flow cytometry analysis revealed a significant increase in the proportion of cells undergoing early- and late-phase apoptosis in a dose- and time-dependent manner after treatment with both compounds (Fig. 3B-C). Consistent with the cytotoxic assay, a significant increase in the apoptosis rate was detectable after 4 h of treatment and became more pronounced after 24 h, with a slight increase at 48 h. The lowest concentrations inducing apoptosis were 7.8 μM for ZOT_5_-1-Me and 15.6 μM for ZOT_5_-1-Et.

To further explore the molecular mechanism underlying ZOT-induced apoptosis, caspase-3 and -7 activation was analysed following 4 h, 24 h, and 48 h of treatment. Caspase3/7 activity was undetectable after 4 h treatment but became evident after 24 h and 48 h in a dose- and time-dependent manner for both compounds (Fig. 3D). The minimum concentrations required to activate caspase-3/7 were 7.8 μM for ZOT_5_-1-Me and 15.6 μM for ZOT_5_-1-Et.

To confirm that ZOTs induce apoptosis in U-87 MG cells through caspase activation, a pan-caspase inhibitor, Z-VAD-FMK, was employed. As expected, pre-treatment with Z-VAD-FMK (10 μM) completely blocked caspase 3/7 activation (Fig. 3E). Moreover, Z-VAD-FMK reduced the apoptotic rate induced by ZOT_5_-1-Me from 32.80 ± 1.15 to 13.19 ± 0.50% and by ZOT_5_-1-Et from 65.08 ± 0.25 to 42.35 ± 0.25%, as evaluated by the Annexin V-FITC/PI assay (Fig. 3F-G). The partial attenuation of cytotoxic effects in the presence of Z-VAD-FMK suggests that ZOTs can trigger both caspase-dependent and caspase-independent cell death pathways.

Our findings demonstrate that ZOT_5_-1-Me and ZOT_5_-1-Et exhibit cytotoxic effects against U-87 MG cells, with ZOT_5_-1-Me showing a more pronounced impact. The induction of cell death by these compounds involves both caspase-dependent and caspase-independent mechanisms.

### ZOT_5_-1-Me and ZOT_5_-1-Et induced intrinsic and extrinsic apoptotic pathways in U-87 MG cells

**Fig. 4:**
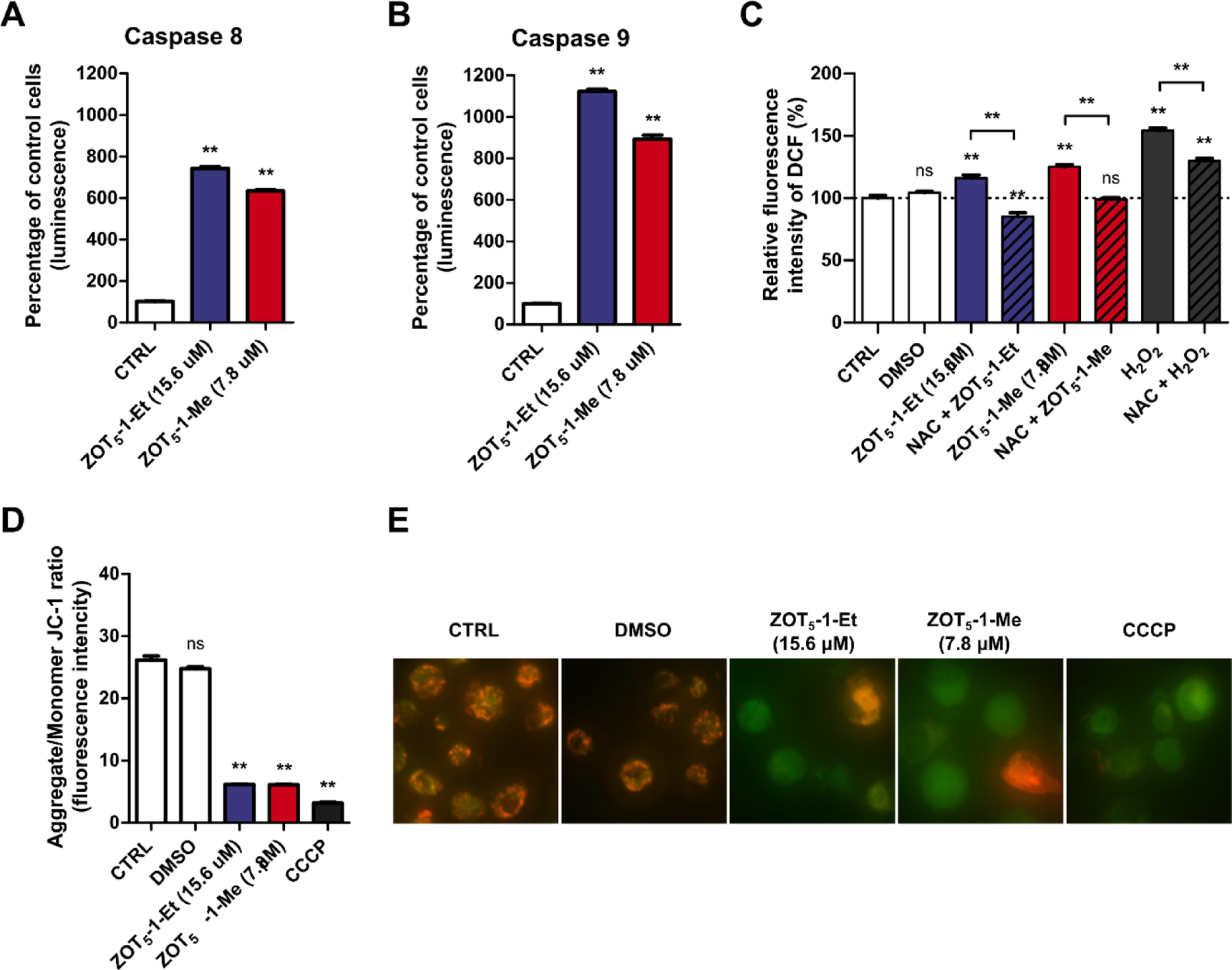
ZOT_5_-1-Me and ZOT_5_-1-Et activated both intrinsic and extrinsic apoptotic pathways in U-87 MG cells. (**A**, **B**) ZOT_5_-1-Me and ZOT_5_-1-Et activated caspase-8 and caspase-9 in U-87 MG after 48 h treatment as evaluated by Caspase-Glo 8 and Caspase-Glo 9 assays, respectively. The data were normalized to control cells (CTRL, 100%, *n* = 6). (**C**) ZOT_5_-1-Me and ZOT_5_-1-Et increased intracellular reactive oxygen species (ROS) in U-87 MG after 2 h treatment as estimated by CM-H_2_DCFDA assay. Hydrogen peroxide (H_2_O_2_) was used as a positive control. (**D**) ZOT_5_-1-Me and ZOT_5_-1-Et reduced mitochondrial membrane potential in U-87 MG after 48 h treatment as assayed by JC-1 staining (*n* = 6). Carbonyl cyanide 3-chlorophenylhydrazone (CCCP, 25 µM, 48 h) was used as a positive control. The value of ΔΨm was expressed as the ratio of JC-1 dimer fluorescence to monomer fluorescence. The data were normalized to control cells (CTRL, 100%, *n* = 6). (**E**) Representative images of JC-1 assay are presented. DMSO was used as a solvent control. Results presented as bar plots with mean ± SEM; ^**^p < 0.01; *ns* – not statistically significant.

Activation of caspases, which drive apoptotic changes, can occur through two distinct pathways: the intrinsic pathway, involving the mitochondria, and the extrinsic pathway, mediated by death receptors. To identify which apoptotic pathways are induced by ZOTs, we analysed the activation of caspase-8 and caspase-9, which are key markers of the extrinsic and intrinsic pathways, respectively. Our results showed that both ZOT_5_-1-Me and ZOT_5_-1-Et activated caspase-8 and caspase-9, suggesting that these compounds trigger apoptosis through a dual mechanism involving both the intrinsic and extrinsic pathways (Fig. 4A-B). To confirm the involvement of the intrinsic pathway, we assessed mitochondrial function by measuring changes in mitochondrial membrane potential (ΔΨm) using the JC-1 assay. The observed loss of ΔΨm further supported the involvement of the intrinsic pathway, as dissipation of ΔΨm is a hallmark of mitochondrial-mediated apoptosis (Fig. 4D-E). Given that mitochondrial dysfunction is often associated with oxidative stress, we also measured ROS levels using the CM-H_2_DCFDA assay. A brief 2-h treatment with ZOT_5_-1-Me and ZOT_5_-1-Et significantly increased ROS levels in a dose-dependent manner, with a more pronounced effect observed for ZOT_5_-1-Me (Fig. 4C). These findings support the notion that both ZOT_5_-1-Me and ZOT_5_-1-Et exert cytotoxic effects via induction of extrinsic and intrinsic apoptotic pathways. Additionally, these findings suggest that oxidative stress may contribute to the induction of apoptosis through mitochondrial dysfunction. The increased ROS could further promote the intrinsic pathway by exacerbating mitochondrial damage and facilitating the release of apoptogenic factors.

### ZOT_5_-1-Me and ZOT_5_-1-Et induced DNA damage, including DNA double-strand breaks in U-87 MG cells

**Fig. 5:**
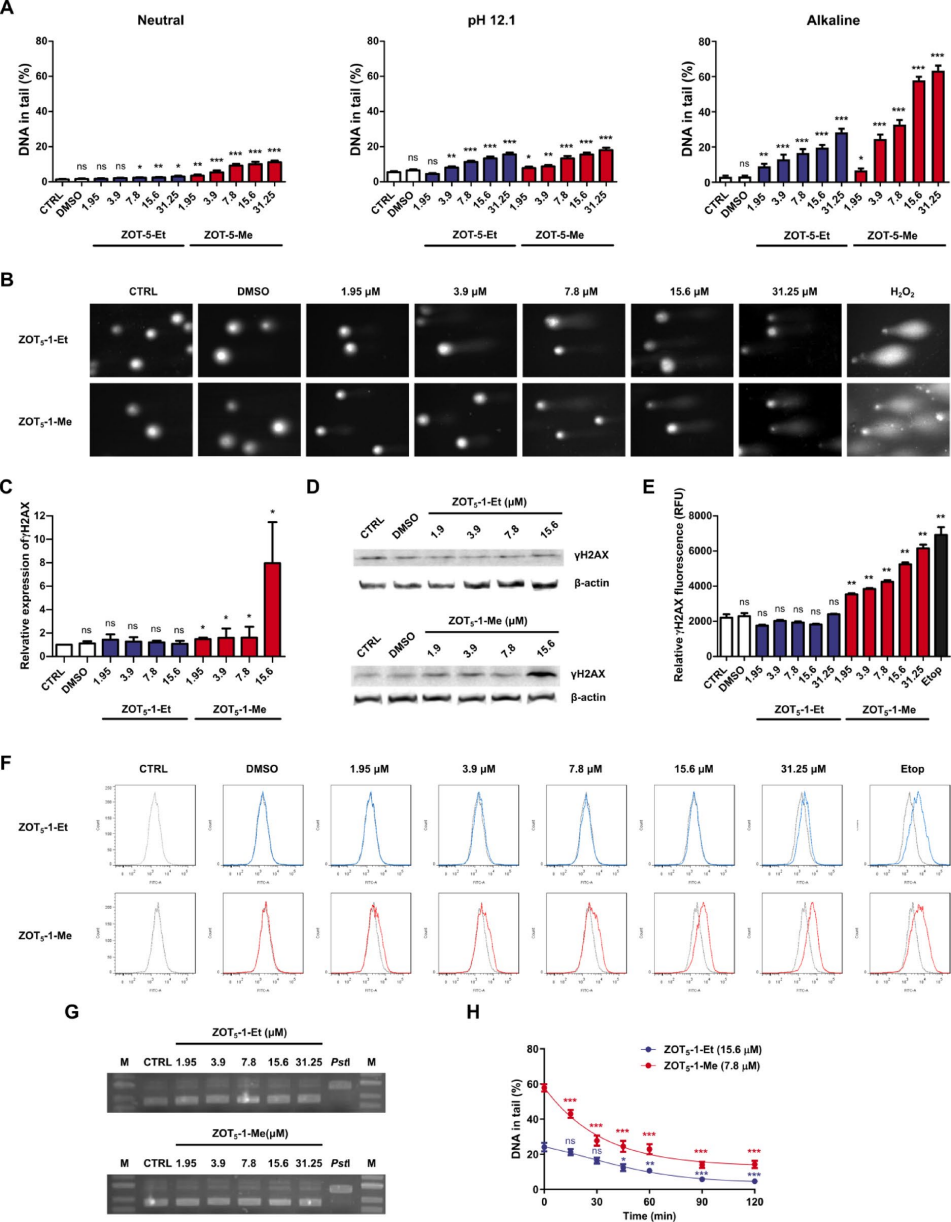
ZOT_5_-1-Me and ZOT_5_-1-Et induced DNA damage, including DNA double-strand breaks in U-87 MG cells. (**A**) ZOT_5_-1-Me and ZOT_5_-1-Et induced DNA damage (percentage of DNA in the comet tail) in U-87 MG cells after 2 h treatment as determined by neutral, pH 12.1, and alkaline versions of the comet assay (*n* = 100). (**B**) Representative images of comets from the alkaline version of the comet assay are presented. (**C**) ZOT_5_-1-Me, but not ZOT_5_-1-E,t induced phosphorylation of histone H2AX (Ser139) in U-87 MG cells after 2 h treatment as assayed by Western blot (*n* = 4). The intensity of bands corresponding to proteins was analysed by densitometry. The results are shown as the fold change of γH2AX levels of treated cells vs. control cells (CTRL). β-actin served as a loading control. (**D**) Representative Western blot images are presented. (**E**) ZOT_5_-1-Me, but not ZOT_5_-1-Et, induced phosphorylation of histone H2AX (Ser139) in U-87 MG cells after 2 h treatment as evaluated by immunofluorescence staining followed by flow cytometry (FACS) quantification (*n* = 6). Etoposide (Etop, 50 µM, 4 h) was used as a positive control. (**F**) Representative FACS histograms are presented. (**G**) ZOT_5_-1-Me and ZOT_5_-1-Et do not induce DNA breaks in isolated supercoiled plasmid as determined by plasmid relaxation assay following 24 h treatment. Lane M – DNA ladder; CTRL – negative control, supercoiled plasmid, *Pst*I – positive linear control (plasmid incubated with *Pst*I restriction enzyme for the induction of DNA double-strand break. (**H**) Time course of repair of DNA damage induced by ZOT_5_-1-Me and ZOT_5_-1-Et in U-87 MG cells after 2 h treatment. After the exposure, the cells were washed and incubated in medium at 37 °C for the indicated time points. The extent of DNA damage at each time point was estimated by the alkaline version of the comet assay (*n* = 100). DMSO was used as a solvent control. Results are presented as bar plots with mean ± SEM; ^*^p < 0.05; ^**^p < 0.01; ^***^p < 0.001, *ns* – not statistically significant.

Given that apoptosis can often result from DNA damage, particularly double-strand breaks (DSBs), we further assessed the DNA-damaging potential of these compounds using the comet assay. A brief 2-h treatment with ZOTs was conducted to evaluate whether DNA damage could serve as a primary mechanism underlying their cytotoxicity. The neutral comet assay revealed that ZOT_5_-1-Me induced DSBs, whereas ZOT_5_-1-Et did not exhibit this effect (Fig. 5A-B). Additionally, the pH 12.1 version of the assay demonstrated the presence of single-strand breaks (SSBs), and the alkaline version identified alkali-labile sites (ALS) as the predominant type of DNA damage induced by both compounds. The induction of DSBs, recognized as the most lethal form of DNA lesion, was further validated by γH2AX analysis. Western blotting (Fig. 5C-D) and flow cytometry (Fig. 5E-F) findings confirmed that ZOT_5_-1-Me, but not ZOT_5_-1-Et, significantly induced DSBs in U-87 MG cells.

To further investigate the ability of ZOT_5_-1-Me and ZOT_5_-1-Et to induce DNA damage, we performed an in vitro cell-free plasmid relaxation assay. This assay evaluates the induction of relaxed and linear forms of plasmid DNA, indicative of SSBs and DSBs, respectively, using gel electrophoresis. Neither ZOT_5_-1-Me nor ZOT_5_-1-Et altered the relative proportions of relaxed and linear plasmid DNA, as determined by their electrophoretic migration profiles. This indicates that both compounds require metabolic activation to exert their genotoxic effects and are incapable of directly inducing DNA breaks in isolated plasmid DNA (Fig. 5G).

Given the extensive and heterogeneous nature of DNA damage induced by ZOT_5_-1-Me and ZOT_5_-1-Et, we further evaluated the capacity of cells to repair these lesions. Using the alkaline version of the comet assay, we monitored the progression of DNA repair over a 2-h recovery period following ZOT treatment, a timeframe sufficient for DSB resolution. DNA damage induced by ZOT_5_-1-Et was fully repaired within this period, with damage levels returning to those observed in untreated controls. In contrast, cells treated with ZOT_5_-1-Me retained significant levels of DNA damage after 2 h of recovery (14.79 ± 2.09), suggesting that ZOT_5_-1-Me may interfere with DNA repair pathways (Fig. 5H).

### ZOT_5_-1-Me and ZOT_5_-1-Et arrested U-87 MG cells in S phase by activating p53-p21 pathway

Unrepaired DNA damage, particularly DSBs, activates the DNA damage response (DDR), which arrests cell cycle at G1/S and G2/M checkpoints to allow time for DNA repair, preventing progression into the S and M phases with damaged DNA and reducing the risk of mutagenesis. Given that i) ZOT_5_-1-Me and ZOT_5_-1-Et induce significant and heterogeneous DNA lesions, ii) ZOT_5_-1-Me generates DSBs and iii) DNA repair pathways failed to resolve ZOT_5_-1-Me-induced DNA lesions within 2 h, we investigated the potential of ZOTs to induce cytostatic effects in greater detail.

**Fig. 6:**
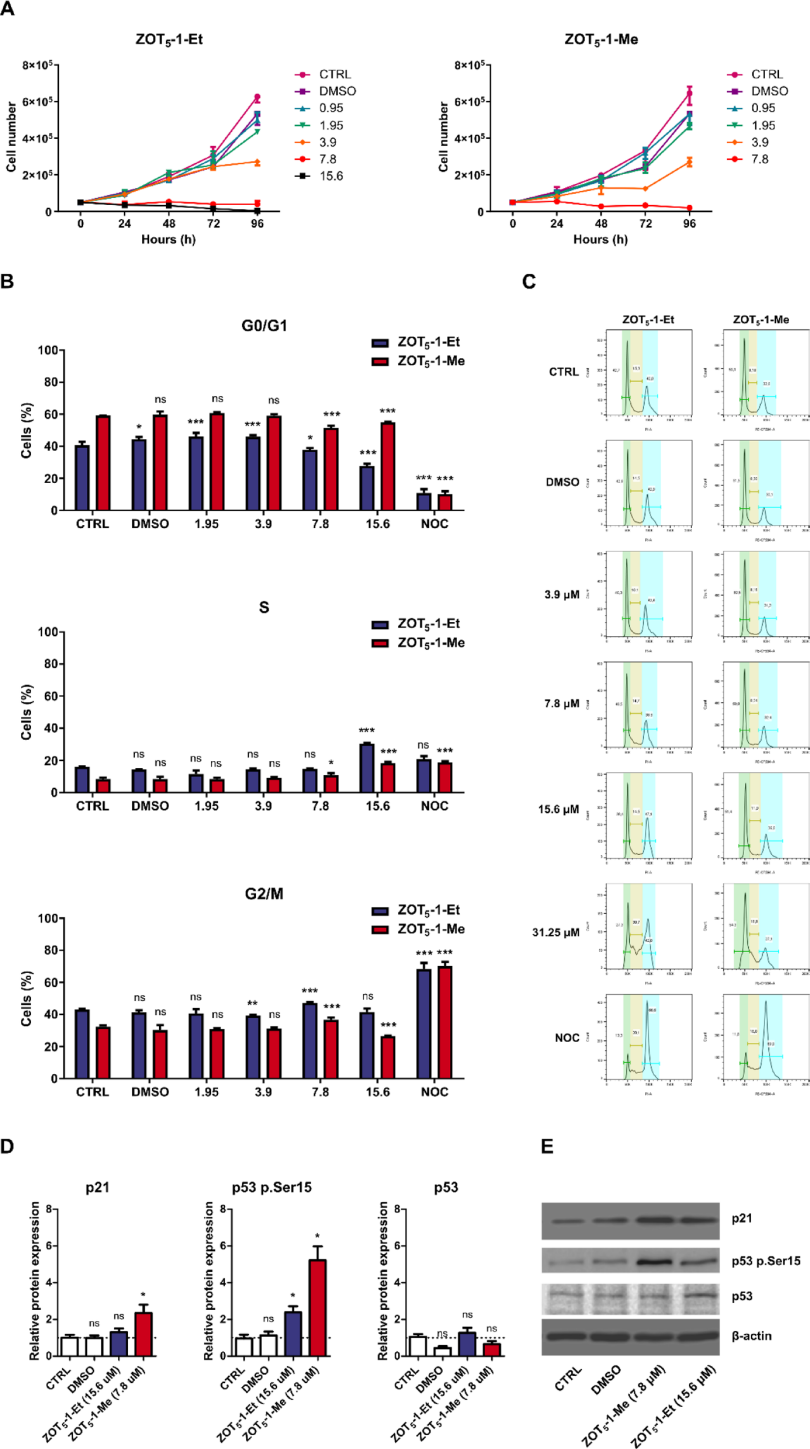
ZOT_5_-1-Me and ZOT_5_-1-Et induced cell cycle arrest in U-87 MG cells. (**A**) Decreased proliferation rates of U-87 MG cells after treatment with an increasing concentration (0.95–15.6 µM) of ZOT_5_-1-Me and ZOT_5_-1-Et for 1–4 days were evaluated by cell proliferation assay. Proliferation curves allowed for the calculation of population doubling times. (**B**) ZOT_5_-1-Me and ZOT_5_-1-Et induced cell cycle arrest in U-87 MG cells after 48 h treatment as determined by PI staining followed by FACS analysis. Nocodazole (NOC, 200 ng/mL, 18 h) was used as a positive control of G2/M arrest. (**C**) Representative FACS dot plots after 48 h treatment with ZOTs are presented. FACS dot plots after 24 h treatments are presented in the Supplementary material. (**D**) ZOT_5_-1-Me and ZOT_5_-1-Et evoked changes to proteins regulating cell cycle, including p21 and phosphorylation of p53 (Ser15), in U-87 MG cells after 48 h treatment as evaluated by Western blot (*n* = 4). The intensity of bands corresponding to proteins was analysed by densitometry. The results are shown as the fold change of protein levels of treated cells vs. control cells (CTRL). β-actin served as a loading control. (**E**) Representative Western blot images are presented. DMSO was used as a solvent control. Results are presented as bar plots with mean ± SEM; ^*^p < 0.05; ^**^p < 0.01; ^***^p < 0.001, *ns* – not statistically significant.

Initially, we evaluated the ability of ZOT_5_-1-Me and ZOT_5_-1-Et to reduce cell proliferation rate and extend PDTs. The treatment with an increasing concentrations of ZOT_5_-1-Me and ZOT_5_-1-Et up to 4 days led to a significant reduction in the cell proliferation rate of U-87 MG cells (Fig. 6A). Notably, proliferation rates dropped below zero at concentrations above 7.8 μM, indicating cytotoxic effects. Calculations at the highest measurable concentrations (3.9 μM) yielded PDTs of 39.4 h for ZOT_5_-1-Me and 39.8 h for ZOT_5_-1-Et, which were significantly longer compared to control cells, which exhibited a PDT of 26.3 h. These findings indicate that both ZOT_5_-1-Me and ZOT_5_-1-Et exert antiproliferative effects in U-87 MG cell line.

To elucidate the underlying mechanisms of ZOT-induced cytostatic effects, we analysed cell cycle distribution via flow cytometry. The treatment with ZOT_5_-1-Me and ZOT_5_-1-Et caused a significant accumulation of cells in the S phase, accompanied by a reduction in the G0/G1 population (Fig. 6B-C). Additionally, the G2/M phase population remained relatively unaffected, suggesting that the primary arrest occurs during DNA synthesis and may be associated with the persistence of DNA damage.

To further investigate the molecular mechanisms underlying the antiproliferative activity of ZOTs, we analysed the expression of cell cycle regulatory proteins with Western blot. We focused on the phosphorylation of p53 at Ser15 and the expression of p21, a potent inhibitor of cell cycle progression. The data revealed that ZOT_5_-1-Me and ZOT_5_-1-Et induced phosphorylation of p53 at Ser15, a marker of DDR activation. As expected, the expression levels of total p53 remained unchanged across treatments. Notably, only ZOT_5_-1-Me significantly upregulated p21 expression, indicating that this compound may have a stronger impact on the p53-p21 axis of cell cycle regulation (Fig. 6D-E).

Taken together, these findings suggest that ZOT_5_-1-Me and ZOT_5_-1-Et exert cytostatic effects by arresting U-87 MG cells in the S phase of the cell cycle. This effect appears to be mediated through the activation of the p53-p21 signalling pathway, primarily through activation of the p53 pathway, with ZOT_5_-1-Me showing a more pronounced effect via p21 upregulation.

### The cytotoxic and genotoxic effects of ZOT_5_-1-Me and ZOT_5_-1-Et are mediated by ROS

**Fig. 7:**
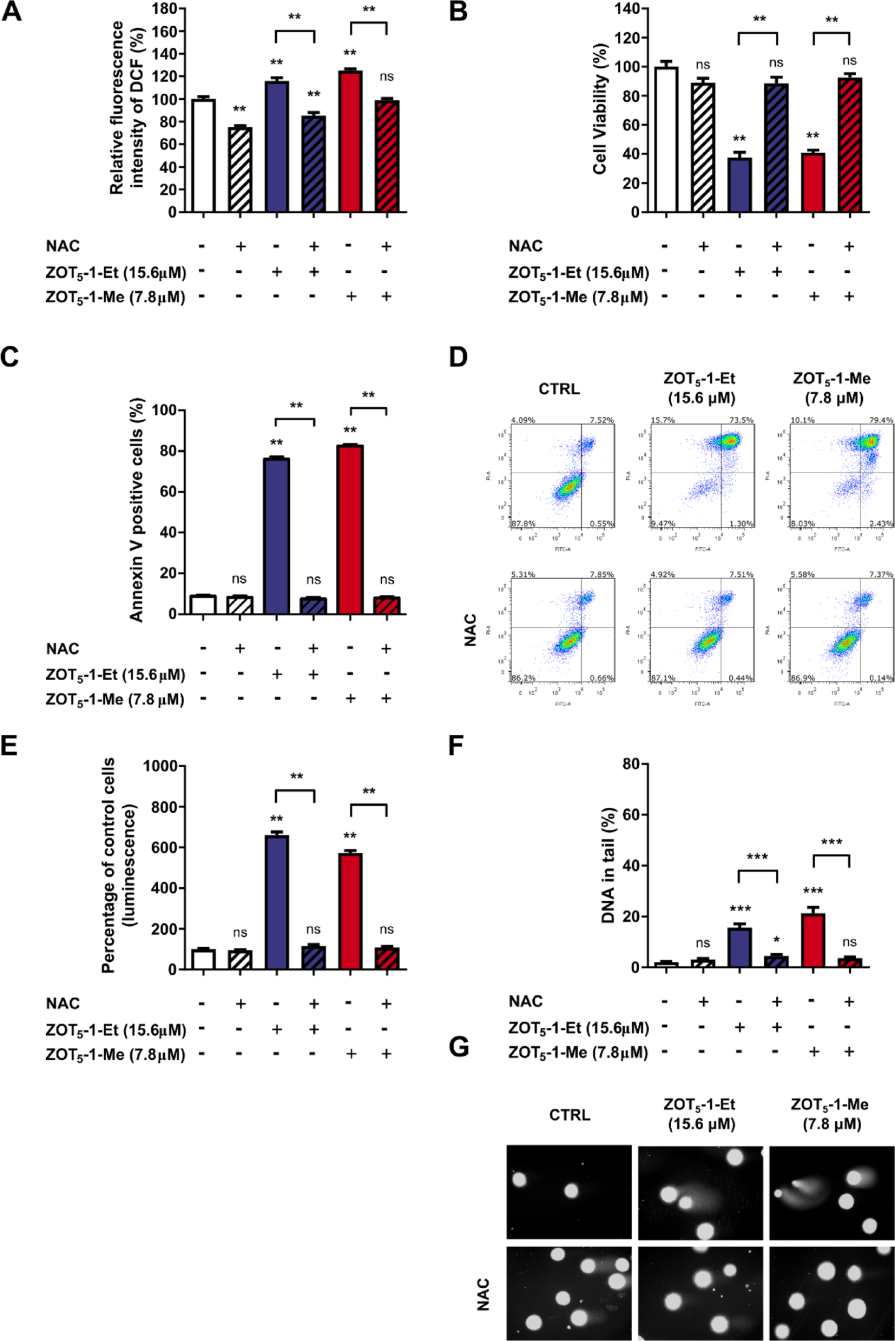
Pre-treatment with ROS scavenger completely blocked the cytotoxic and genotoxic effect of ZOT_5_-1-Me and ZOT_5_-1-Et in U-87 MG cells. (**A**) Pre-incubation with *N*-acetyl-l-cysteine (NAC, 1 mM, 1 h) decreased intracellular reactive oxygen species (ROS) induced by ZOT_5_-1-Me and ZOT_5_-1-Et in U-87 MG as estimated by CM-H_2_DCFDA assay. The data were normalized to control cells (CTRL, 100%, *n* = 6). (**B**) Pre-incubation with NAC restored cell viability of U-87 MG cells after 48 h treatment with ZOT_5_-1-Me and ZOT_5_-1-Et as evaluated by CCK-8 assay. The data were normalized to control cells (CTRL, 100%, *n* = 6). (**C**) Pre-incubation with NAC reduced the externalisation of phosphatidylserine in U-87 MG cells as evaluated by Annexin V/PI assay followed by flow cytometry (FACS) quantification (*n* = 6). (**D**) Representative FACS dot plots after 48 h treatment with ZOTs are presented with the indicated percentages of necrotic (Q1), late-apoptotic (Q2), early-apoptotic (Q3), and viable cells (Q4). Apoptosis was presented as a percentage of Annexin V-positive cells (Q2 + Q3). (**E**) Pre-incubation with NAC diminished ZOT_5_-1-Me and ZOT_5_-1-Et-evoked apoptosis in U-87 MG cells as determined by Caspase-Glo 3/7 assay (*n* = 6). The data were normalized to control cells (CTRL, 100%, *n* = 6). (**F**) Pre-incubation with NAC decreased DNA damage (percentage of DNA in the comet tail) induced by ZOT_5_-1-Me and ZOT_5_-1-Et in U-87 MG cells as determined by the alkaline versions of comet assay (*n* = 100). (**G**) Representative images of comets from the alkaline version of the comet assay are presented. Results are presented as bar plots with mean ± SEM; ^*^p < 0.05; ^**^p < 0.01; ^***^p < 0.001, *ns* – not statistically significant.

ZOT_5_-1-Me and ZOT_5_-1-Et were demonstrated to induce ROS accumulation and mitochondrial damage (Fig. 4C-E), suggesting that their cytotoxic and genotoxic effects might be mediated through ROS upregulation. To explore the role of ROS in the mechanism of action of these compounds, we utilized *N*-acetyl-l-cysteine (NAC), a potent ROS scavenger. Cells were pre-incubated with NAC (1 mM, 1 h) prior to the treatment with ZOTs. NAC pre-treatment effectively suppressed ROS production induced by both ZOT_5_-1-Me and ZOT_5_-1-Et (Fig. 7A). Furthermore, NAC completely abrogated the cytotoxic effects of these compounds, as evidenced by the restoration of cell viability levels comparable to untreated controls (Fig. 7B). This protective effect was corroborated by the suppression of apoptotic markers, including Annexin V externalisation (Fig. 7C-D) and caspase 3/7 activation (Fig. 7E). Additionally, NAC mitigated the genotoxic effects of ZOT_5_-1-Me and ZOT_5_-1-Et, as demonstrated by the absence of detectable DNA damage (Fig. 7F-G). Overall, these results support a model in which ZOT_5_-1-Me and ZOT_5_-1-Et induce apoptotic cell death with oxidative stress potentially serving as a key trigger in these processes.

## Discussion

GBM is among the most aggressive and lethal primary brain tumours^1^. Despite multimodal treatment strategies, long-term survival remains limited, with median survival ranging from 14.6 to 21.1 months^4^. The management of GBM is hampered by several challenges, including tumour heterogeneity, the presence of therapy-resistant glioma stem cells, the highly infiltrative growth pattern of tumour, primary and acquired drug resistance, and BBB, which restricts drug delivery^7,8,27–29^. These factors significantly complicate the development of effective therapies.

The majority of approved anticancer drugs are unable to cross the BBB, reducing therapeutic options. U.S. Food and Drug Administration (FDA) has approved only a few treatments for GBM, including the chemotherapeutics lomustine (CCNU, 1976), carmustine (BCNU, 1977), and TMZ (2005), as well as anti-vascular endothelial growth factor (VEGF) antibody bevacizumab (2009), and tumour treating fields (TTF, 2011)^30,31^. Among these, TMZ can cross the BBB and shows bioavailability of 98% when orally administered^32–34^. Although TMZ is the first-line chemotherapy for GBM, its effectiveness is limited, particularly against recurrent tumours^35,36^. Lomustine and carmustine, despite their ability to penetrate the BBB, offer limited benefits for GBM patients^37,38^. On the other hand, bevacizumab – a monoclonal antibody that inhibits angiogenesis by targeting VEGF-A – cannot penetrate the BBB, and thus its efficacy is limited, resulting in no improvement in the overall survival of GBM patients^39,40^.

Given that no current treatment regimen for GBM is curative, the National Comprehensive Cancer Network (NCCN) recommends clinical trials as the preferred option for eligible patients, reflecting the need for innovative approaches^41^. Currently, there are 286 ongoing clinical trials focused on GBM, underscoring the scientific community’s recognition of the urgent need for new therapies^42^.

Although numerous compounds have demonstrated cytotoxic effects against GBM cells in vitro, translating these preclinical findings into clinical success has proven exceptionally rare. Nevertheless, novel agents are currently being developed, either as monotherapies or in combination regimens^28,36,43^. Most therapeutic advances in GBM therapy have been hampered due to the restricted permeability across the BBB, which permits only small (< 500 Da and < 400 nm) and lipophilic molecules to pass diffusely across, while larger or hydrophilic compounds are typically excluded^8,44,45^. Consequently, GBM therapy has seen little advancement in the past two decades, with the Stupp protocol remaining the standard of care^4,5^. The simplest polyfluoroalkyl phosphonates were designed as small molecular weight (< 250 Da) anticancer candidates with the potential to cross the BBB^19^. These compounds were initially screened against GBM cells, and the most cytotoxic compounds, ZOT_5_-1-Me and ZOT_5_-1-Et, were selected for further analysis. To expand our knowledge of the biological activity of the simplest polyfluoroalkyl phosphonates, this study focuses on elucidating the molecular mechanism of ZOT_5_-1-Me and ZOT_5_-1-Et, as promising candidates for chemotherapeutics against GBM.

### Mechanisms of action of ZOT compounds

In this study, we first examined the anticancer potential of ZOT_5_-1-Me and ZOT_5_-1-Et in the U-87 MG cell line. We found that ZOT_5_-1-Me and ZOT_5_-1-Et induced cell death through both intrinsic and extrinsic apoptotic pathways. This dual activation is a critical finding, as it suggests the compounds can trigger apoptosis from multiple cellular signals. Apoptosis is often dysregulated in cancer cells, allowing them to evade normal cell death mechanisms. These compounds can effectively decrease tumour cell survival by activating initiator caspase 3/7, and executive caspase-8 and caspase-9. Additionally, pan-caspase inhibitor Z-VAD-FMK has not completely prevented cell death, although caspase activity was completely inhibited (Fig. 3E-G), indicating the activation of caspase-independent cell death pathways.

DNA is among the principal targets of many anticancer drugs. Multiple DDR inhibitors are now being tested in clinical trials for GBM^46^. All clinically used FDA-approved chemotherapeutics against GBM belong to DNA alkylating agents. The cytotoxic effect of TMZ is primarily exerted by methylation of *O*^*6*^ position of guanine (*O*^*6*^-meG) which escapes *O*^*6*^-meG-DNA-methyl transferase (MGMT) repair resulting in futile cycles of mismatch repair leading to DSBs and triggering cell cycle arrest and apoptosis^32^. MGMT down-regulation is observed in 40% of GBM patients due to *MGMT* gene promoter methylation. Not all GBM patients respond to TMZ, and drug resistance has been correlated to *MGMT* overexpression and/or malfunctioning mismatch repair (MMR)^47^. Lomustine and carmustine are bifunctional DNA alkylating agents and give rise to the formation of interstrand crosslinks (ICLs), which are critical cytotoxic DNA lesions that block DNA replication and transcription^48^. Besides ICLs, DSBs are considered the most lethal form of DNA damage and a primary cause of cell death. Recently, SSBs are also becoming recognized as important lesions for lethality^46^. We have demonstrated that both ZOT_5_-1-Me and ZOT_5_-1-Et induced DNA damage, including SSBs and ALS, in the U-87 MG cell line (Fig. 5A-B). Interestingly, ZOT_5_-1-Me was effective at inducing DSBs compared to ZOT_5_-1-Et, indicating potential differences in their mechanisms of action (Fig. 5). Both compounds appear to act as prodrugs, requiring metabolic activation to induce DNA damage, as they were chemically inert in their initial state during plasmid relaxation assays (Fig. 5G).

Targeting the cell cycle remains a mainstay in the treatment of cancer. All FDA-approved chemotherapeutics against GBM are cytostatic drugs inhibiting the proliferation of GBM cells, which are characterized by rapid division and growth. Both ZOT_5_-1-Me and ZOT_5_-1-Et had an antiproliferative effect on U-87 MG cells, which was associated with cell cycle arrest at the S phase (Fig. 6A-C). The ability of ZOT_5_-1-Me and ZOT_5_-1-Et to arrest the cell cycle correlated with their capacity to induce DNA damage. Additionally, both compounds induced phosphorylation of p53, a tumour suppressor protein, and ZOT_5_-1-Me increased the expression of p21, a cyclin-dependent kinase inhibitor, indicating that ZOTs at least partially arrest cell cycle via p53-p21 signalling pathway (Fig. 6D-E).

ZOT_5_-1-Me and ZOT_5_-1-Et significantly increased intracellular ROS levels in U-87 MG cells, with a concentration-dependent relationship, suggesting oxidative stress as a potential mechanism of cytotoxicity (Fig. 4C). Indeed, the cytotoxic and genotoxic effects of ZOT_5_-1-Me and ZOT_5_-1-Et were strongly dependent on their ability to elevate ROS (Fig. 7). Redox imbalance plays a complex and dualistic role in GBM, contributing to both tumour progression and resistance to therapy^7,49,50^. In this context, both antioxidant and pro-oxidant therapeutic strategies have been explored in GBM. Antioxidants, particularly at high or pharmacological doses, can paradoxically exert pro-oxidant effects by inducing oxidative stress sufficient to trigger cancer cell death, while potentially reducing non-specific toxicity associated with chemotherapy^50^. A prominent example is ascorbate (pharmacological vitamin C), which exhibited pro-oxidant activity at high concentrations. A phase II clinical trial is currently ongoing to assess the efficacy of high-dose ascorbate in combination with radiotherapy and adjuvant chemotherapy with TMZ^51–54^. Nevertheless, reducing ROS levels within tumours can be a double-edged sword. Antioxidants may inadvertently protect cancer cells from ROS-mediated cell death, thereby attenuating the efficacy of chemo-radiation, as demonstrated in some preclinical studies^10,55^. Conversely, pro-oxidant therapies aim to increase ROS levels beyond a critical threshold, selectively inducing cancer cell death. Standard therapies such as radiotherapy and chemotherapy rely in part on ROS-mediated mechanisms to achieve their cytotoxic effects. Although preclinical findings are promising, pro-oxidants are yet to progress through the clinical trial phase^10,12–14^. Given the importance of ROS in mediating the cytotoxic effects of ZOTs, combining these compounds with conventional therapies that further elevate oxidative stress may enhance their therapeutic efficacy as noted for other pro-oxidants^56–58^. Nonetheless, this approach requires careful consideration of potential off-target effects on surrounding normal brain tissue^50^. Collectively, the available evidence suggests that a therapeutic window exists for ROS-modulating strategies in GBM, either as monotherapy or in combination with conventional treatments. While ZOTs exhibit strong anticancer potential through ROS generation, successful translation into clinical practice will depend on achieving a delicate balance – maximizing cytotoxic effects within GBM cells while preserving the integrity and function of healthy brain tissue.

### Molecular determinants of ZOT sensitivity in glioma

To elucidate the relationship between genetic alterations and the sensitivity of glioma cell lines to the novel compounds ZOT_5_-1-Me and ZOT_5_-1-Et, we analysed the mutation status of key biomarkers, including *TP53*, *PTEN*, *CDKN2A*, and *IDH*, across seven glioma cell lines: SF-268, SNB-19, SNB-75, U251, SF-539, SF-295, and U-87 MG (Table 2). Differential sensitivity was observed, with higher sensitivity towards GBM cell lines SF-539, SF-295, and U-87 MG, as well as the U251 astrocytoma cell line, and lower sensitivity towards astrocytoma SF-268 and SNB-19, and GBM SNB-75 cell lines (Fig. 2A, Fig. 3A, Table 1).

**Table 2:** Molecular status of genetic biomarkers in glioma cell lines^59–63^.

Cell lines	*CDKN2A*	*IDH*	*PTEN*	*TP53*	Glioma type/subtype
SF-268	Mutated	Mutated*	Wild-type	Mutated	Astrocytoma
SNB-19	Mutated	No data	Mutated	Mutated	Astrocytoma
SNB-75	Wild-type	Wild-type*	Wild-type	Mutated	Glioblastoma
U251	Mutated	Wild-type	Mutated	Mutated	Astrocytoma
SF-539	Wild-type	Wild-type*	Mutated	Mutated	Glioblastoma
SF-295	Mutated	No data	Mutated	Mutated	Glioblastoma
U-87 MG	Mutated	Wild-type	Mutated	Wild-type	Glioblastoma

* according to COSMIC, CCLE, Cell model passports, and Cellosaurus database.

Both *PTEN* and *TP53* are frequently mutated in GBM, contributing to aggressive behaviour, treatment resistance, and poor prognosis^64–67^. Among the analysed cell lines, only U-87 MG retained wild-type *TP53*, which corresponded with the highest cytotoxic response to ZOT_5_-1-Me and ZOT_5_-1-Et (Fig. 3A, Table 2), indicating that *TP53* status may influence drug sensitivity. ZOT efficacy was primarily influenced by *PTEN* mutation status, with *PTEN-*deficient cell lines—particularly SF-539—showing heightened sensitivity, while cell lines with mutated *TP53* and intact *PTEN*—SF-268, SNB-75—were less sensitive (Fig. 2A, Table 1–2). In U-87 MG, the synergy between functional p53 and PTEN loss could lead to the improved cytotoxic outcomes (Fig. 3A, Table 2). Indeed, PTEN is known to cooperate with p53 in the repair of DSBs, a mechanism potentially leveraged by ZOT activity (Fig. 5H)^59^. These results suggest that ZOTs could be most effective in GBM cells with wild-type *TP53* and mutated *PTEN*, a genotype common in IDH-wildtype GBM (~ 90% of adult gliomas)^68^. Conversely, *TP53*-mutant/*PTEN*-intact cells were less sensitive, indicating limited efficacy in other adult-type diffuse gliomas (Fig. 2A, Table 1–2)^69^. Sensitivity was further modulated by mutations in genes like *CDKN2A* and *IDH*, suggesting a multifactorial basis for ZOT responsiveness (Fig. 2A, 3A; Table 1–2). These insights highlight the need for molecular profiling and potential combination therapies in less responsive cases.

### Implications for clinical application

The ability of ZOT_5_-1-Me and ZOT_5_-1-Et to induce cell death through multiple pathways, generate ROS, cause DNA damage, and cell cycle arrest in U-87 MG cells suggests that they hold promise as therapeutic agents for GBM. Their ability to activate a few cell death pathways is particularly significant, as they could overcome some of the primary resistance mechanisms that have hindered the efficacy of other treatments like TMZ. Moreover, the study highlights the potential for ZOTs to be used in combination with other therapies. Given the promising role of ROS in mediating the cytotoxic effects of ZOTs, their combination with agents that further promote oxidative stress in GBM cells may enhance their cytotoxic potential. Additionally, their use alongside traditional DNA-damaging agents like TMZ could further disrupt GBM cell survival by overwhelming their DNA repair mechanisms.

While the results are promising, several limitations warrant further investigation. Firstly, the experiments were conducted in vitro using established GBM cell lines, which may not fully capture the heterogeneity of patient tumours. Moreover, detailed analyses were restricted to the U-87 MG cell line, selected as the most sensitive model for in-depth mechanistic studies, and future work will therefore extend to resistant lines and primary GBM cultures to more accurately capture tumour heterogeneity and mechanisms of therapy resistance. A further limitation is the absence of a non-tumoural neural control cell line, which would help determine whether the observed effects are preferentially directed against transformed cells. Although our previous work included cytotoxicity screening in peripheral blood mononuclear cells (PBMCs) as a representative non-tumoural model, additional data on non-neoplastic neural cells are needed to strengthen the safety assessment^19^. Additionally, employing transwell assays with brain endothelial cells could provide a more physiologically relevant model to confirm the ability of ZOTs to penetrate the BBB. In vivo studies using animal models of GBM are also essential to evaluate the compounds’ capacity to cross the BBB, validate their therapeutic potential within the complex tumour microenvironment, and refine their clinical applications. Finally, comprehensive assessments of the long-term toxicity and pharmacokinetics of ZOT_5_-1-Me and ZOT_5_-1-Et are necessary to establish their safety profile and identify potential side effects in a clinical setting.

Future research should also focus on understanding the precise molecular mechanisms that differentiate ZOT_5_-1-Me and ZOT_5_-1-Et, particularly their differential effects on DSBs. Further exploration of these mechanisms could lead to the optimization of these compounds for enhanced efficacy against GBM. Moreover, the potential for these compounds to synergize with existing therapies, including TMZ and radiotherapy, should be explored in preclinical models.

## Conclusion

**Fig. 8:**
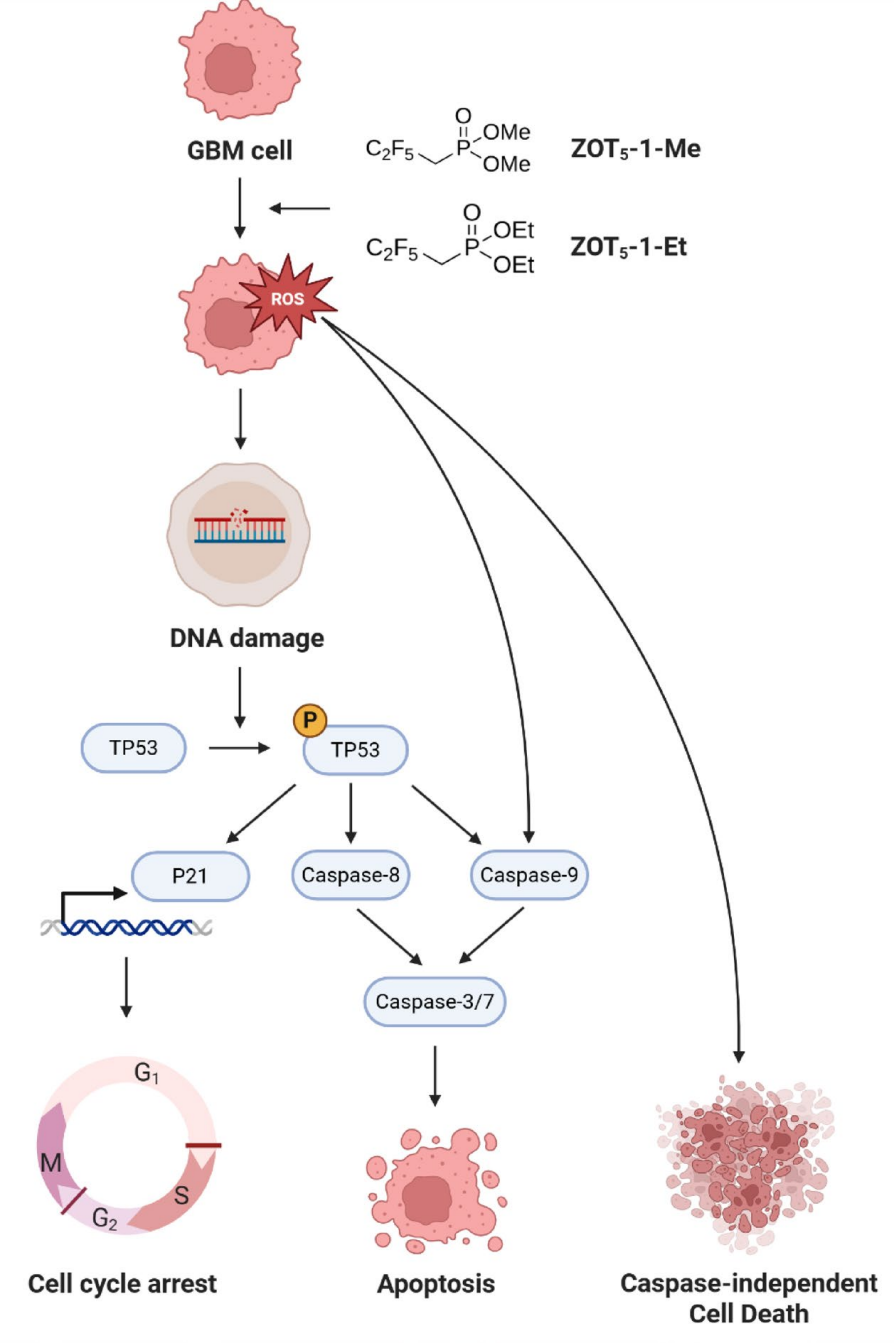
Proposed molecular mechanism underlying anticancer activity of ZOT_5_-1-Me and ZOT_5_-1-Et in glioblastoma (GBM) cells. ZOT_5_-1-Me and ZOT_5_-1-Et elevate the level of reactive oxygen species (ROS) in GBM cells, causing DNA damage and subsequent activation of TP53 pathway. Phosphorylated TP53 upregulates the expression of p21, resulting in cell cycle arrest at G1/S and G2/M checkpoints, and initiates apoptosis through intrinsic (caspase-9) pathway. Additionally, ZOT_5_-1-Me and ZOT_5_-1-Et can trigger extrinsic (caspase-8) apoptotic pathway and caspase-independent cell death pathways (created in BioRender. Wozniak, K. (2025) https://BioRender.com/35yqjr4).

In conclusion, ZOT_5_-1-Me and ZOT_5_-1-Et represent promising new candidates for GBM therapy. Their ability to induce cell death through multiple pathways, generate ROS, and cause DNA damage and cell cycle arrest in GBM cells highlights their potential to overcome some of the most significant challenges in GBM treatment (Fig. 8). However, further studies are necessary to validate these findings in vivo and to fully explore their therapeutic potential in combination with existing treatments. If successful, these compounds could represent a significant advancement in the treatment of GBM, offering new hope for patients with this devastating disease.

## Supplementary Information

Below is the link to the electronic supplementary material.

Supplementary Material 1

## Data Availability

The data generated in this study are available at Zenodo (10.5281/zenodo.16272119). Drug response profiles obtained in this study are available at NCI/DTP repository (https://dtp.cancer.gov/dtpstandard/dwindex/index.jsp); at NSC 835966 (ZOT_5_-1-Me; https://dtp.cancer.gov/dtpstandard/servlet/dwindex?searchtype=NSC&searchlist=835966) and NSC 835967 (ZOT_5_-1-Et, https://dtp.cancer.gov/dtpstandard/servlet/dwindex?searchtype=NSC&searchlist=835967).

## Declarations

### Generative AI and AI-assisted technologies in the writing process

During the preparation of this work the author(s) used chat.gpt in order to improve language and readability. After using this tool/service, the author(s) reviewed and edited the content as needed and take(s) full responsibility for the content of the publication.
