# Supplementary material for: Small molecular weight polyfluoroalkyl phosphonates induce ROS-mediated cytotoxicity in glioblastoma cells: a molecular mechanism study
Source: Sci Rep. 2025 Nov 6;15:38896. doi: 10.1038/s41598-025-22754-0 (PMC12592489; doi:10.1038/s41598-025-22754-0)
Supplement: Supplementary file 1 — Supplementary Material 1 [file 41598_2025_22754_MOESM1_ESM.pdf]

Small Molecular Weight Polyfluoroalkyl Phosphonates Induce ROS-Mediated Cytotoxicity in Glioblastoma: A Molecular Mechanism Study

Patryk Wołodkiewicz[1,2], Michał Juszczak[1], Paweł Tokarz[3], Katarzyna Woźniak[1], Paulina Tokarz[1]*

[1] University of Lodz, Faculty of Biology and Environmental Protection, Department of Molecular Genetics, Pomorska 141/143, 90-236, Lodz, Poland

[2] University of Lodz, University of Lodz Doctoral School of Exact and Natural Sciences, Matejki 21/23, 90-237 Lodz, Poland

[3]University of Lodz, Faculty of Chemistry, Laboratory of Molecular Spectroscopy, Tamka 12, 91-403, Lodz, Poland

Address for correspondence: Paulina Tokarz, e-mail: paulina.tokarz@biol.uni.lodz.pl.

Table of Contents:

Figure S1. ZOT_5-1-Me and ZOT_5-1-Et induced cell death and apoptosis in U-87 MG cells after 4 h and 24 h treatment.

Figure S2. Raw microscope images of JC-1 staining from Fig. 4E.

Figure S3. Raw Western blot membranes images from Fig. 5D.

Figure S4. Raw plasmid relaxation assay gel images from Fig. 5G.

Figure S5. ZOT_5-1-Me and ZOT_5-1-Et induced cell cycle arrest in U-87 MG cells after 24 h treatment.

Figure S6. Raw Western blot membranes images from Fig. 6E.

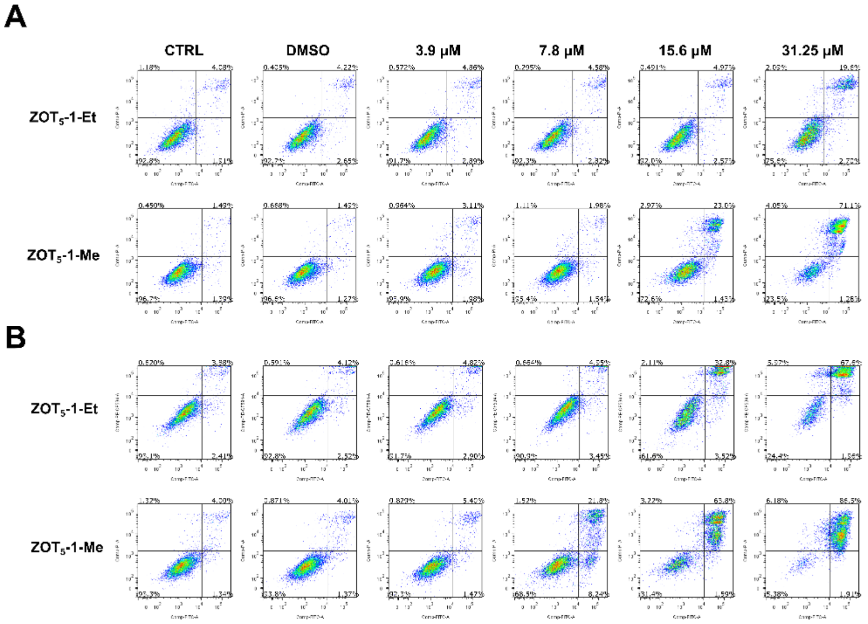

Fig. S1. ZOT_5-1-Me and ZOT_5-1-Et induced cell death and apoptosis in U-87 MG cells. ZOT_5-1-Me and ZOT_5-1-Et induced externalisation of phosphatidylserine in U-87 MG cells as evaluated by Annexin V/PI assay followed by flow cytometry (FACS) quantification (n = 6). Representative FACS dot plots after 4 h (**A**) and 24 h (**B**) treatment with ZOTs are presented with the indicated percentages of necrotic (Q1), late-apoptotic (Q2), early-apoptotic (Q3), and viable cells (Q4).

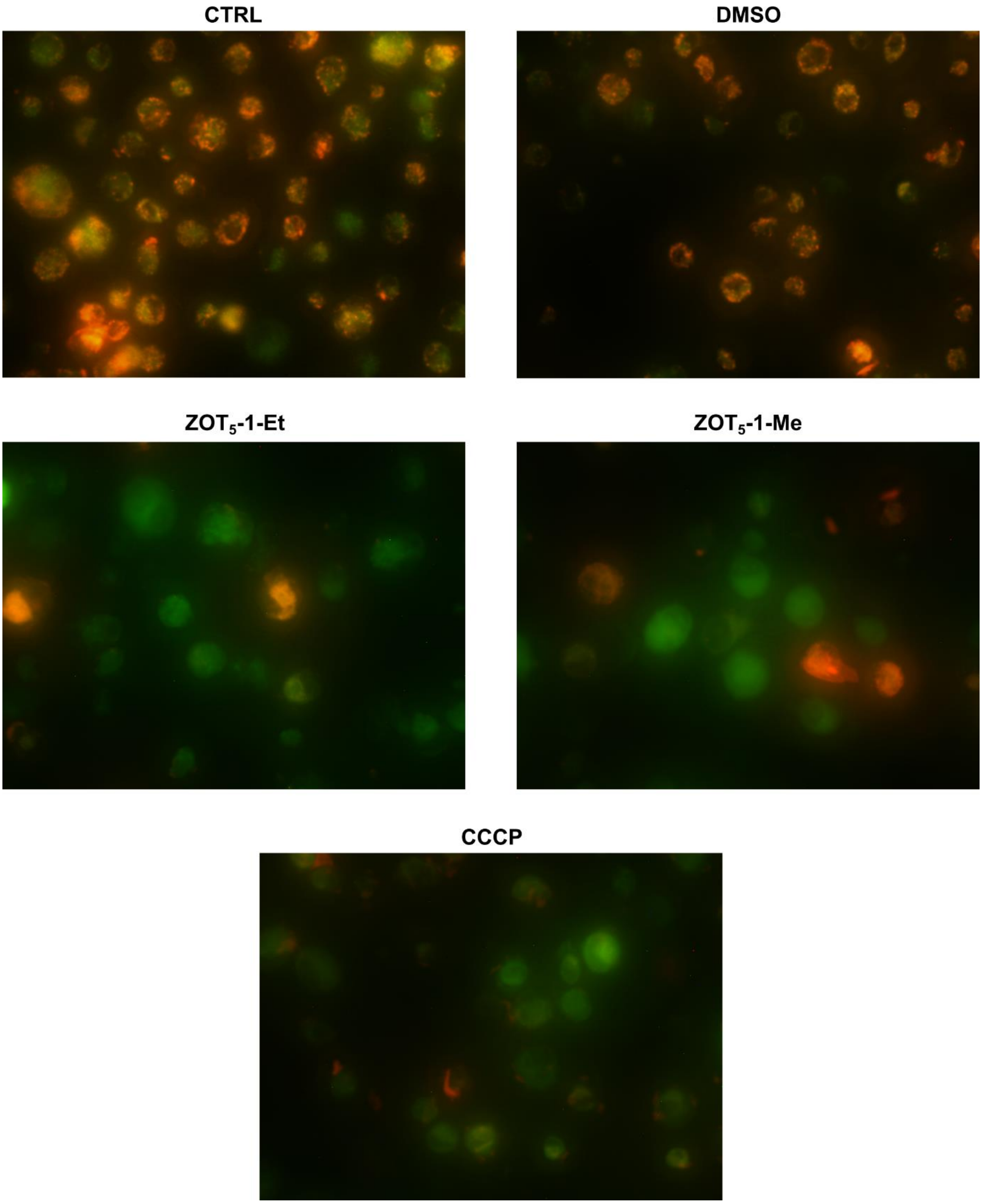

Fig. S2. Raw microscope images of JC-1 staining from Fig. 4E. ZOT$_5$-1-Me and ZOT$_5$-1-Et declined mitochondrial membrane potential in U-87 MG after 48 h treatment as assayed by JC-1 staining. Carbonyl cyanide 3-chlorophenylhydrazone (CCCP) was used as a positive control. DMSO was used as a solvent control.

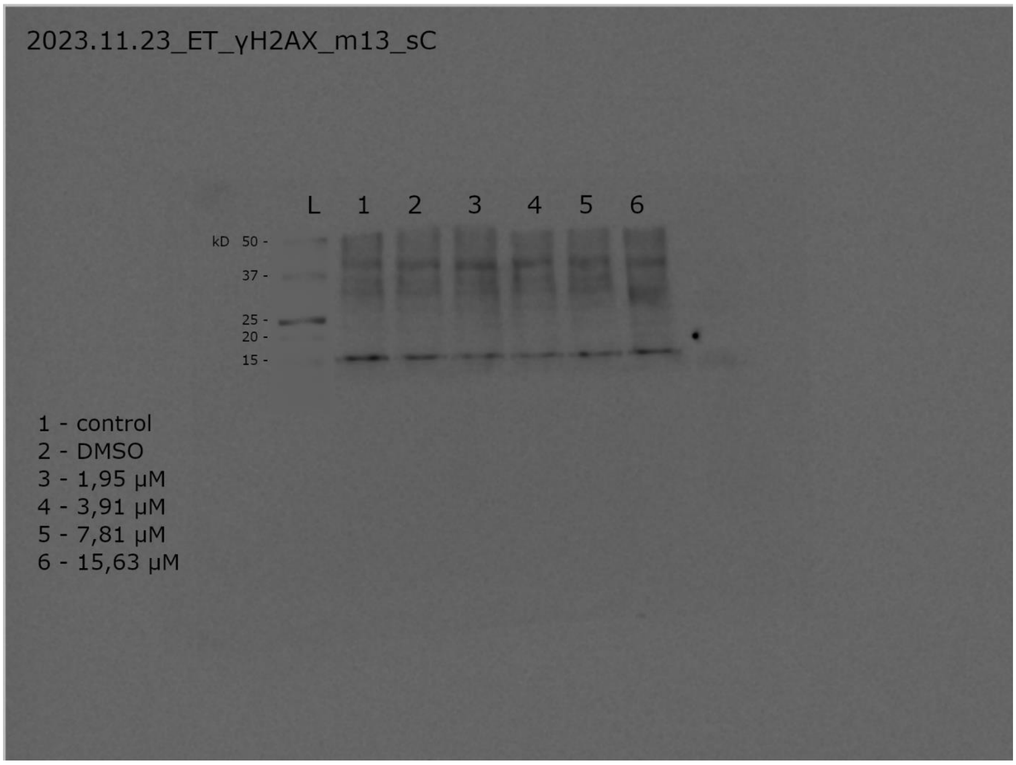
2023.11.23_ET_γH2AX_m13_sC
L 1 2 3 4 5 6
kD 50 -
37 -
25 -
20 -
15 -
1 - control
2 - DMSO
3 - 1,95 μM
4 - 3,91 μM
5 - 7,81 μM
6 - 15,63 μM

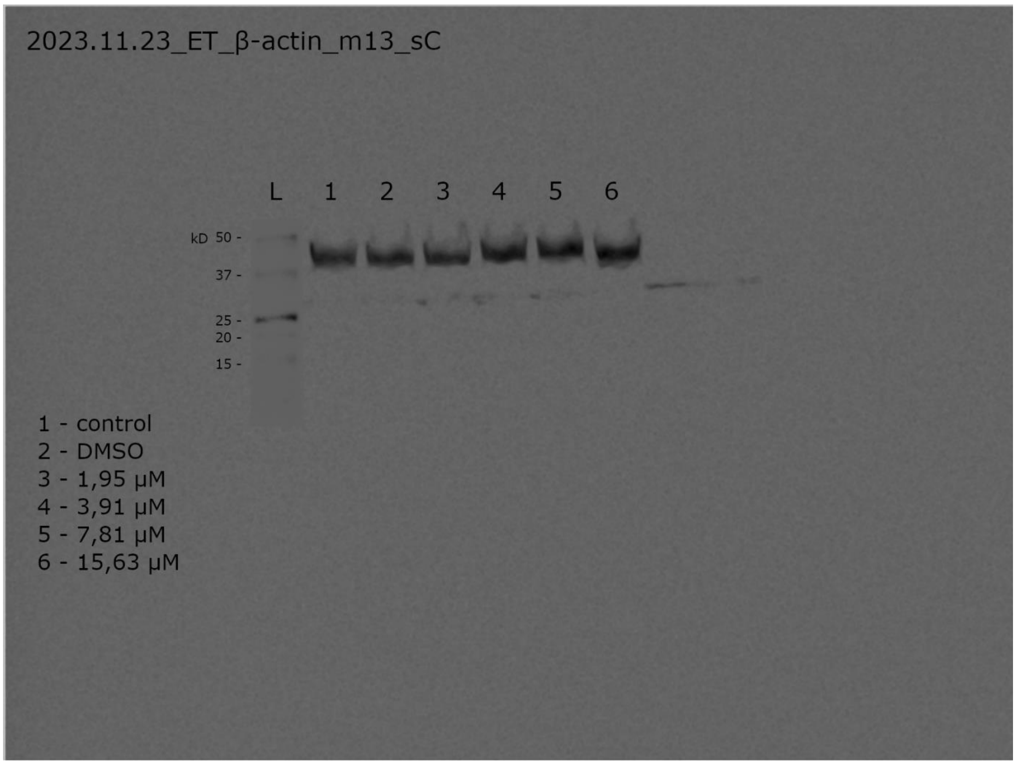
2023.11.23_ET_β-actin_m13_sC
L 1 2 3 4 5 6
kD 50 -
37 -
25 -
20 -
15 -
1 - control
2 - DMSO
3 - 1,95 μM
4 - 3,91 μM
5 - 7,81 μM
6 - 15,63 μM

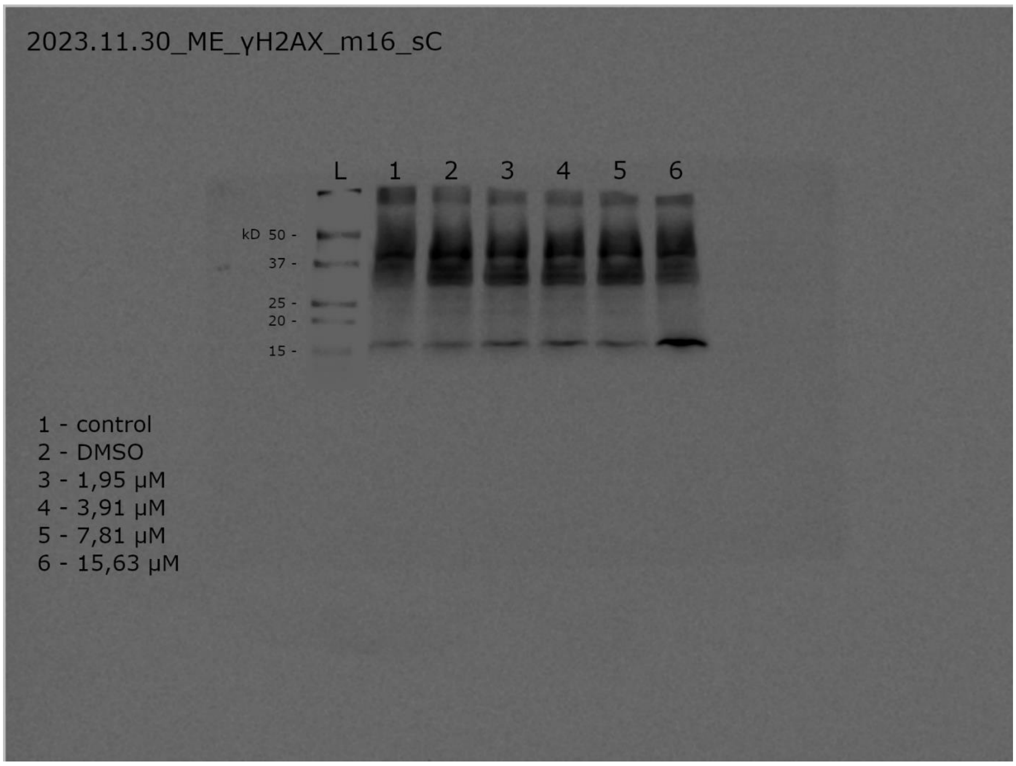

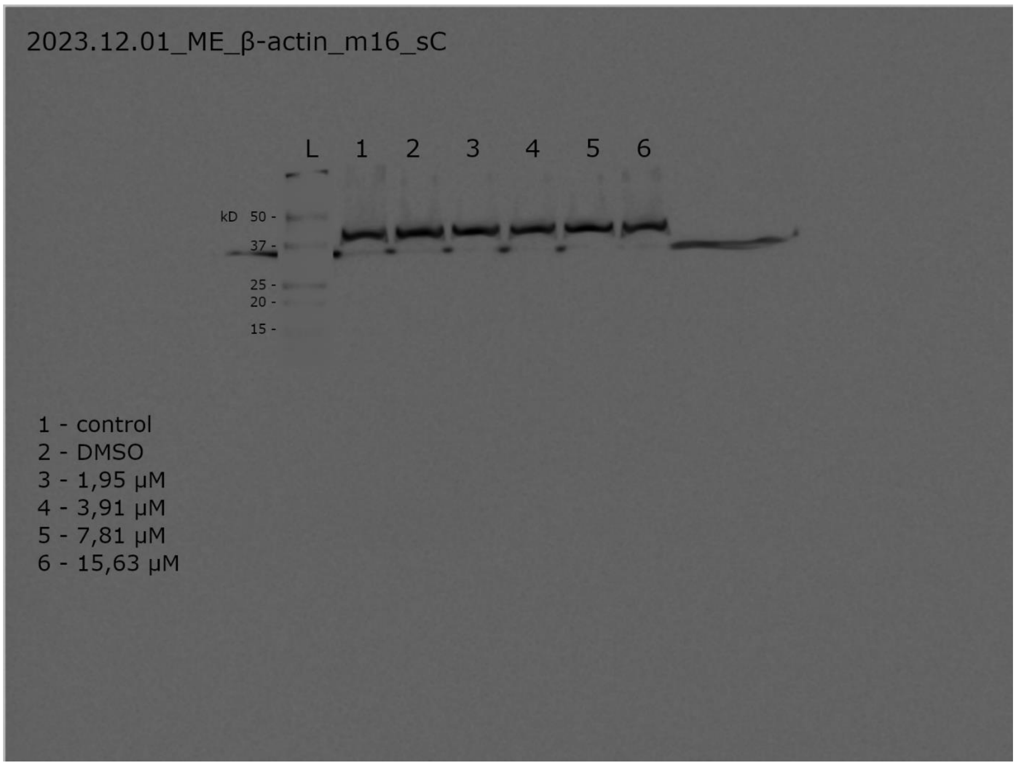

Figure S3. Raw Western blot membranes images from Fig. 5D. ZOT_5-1-Me and ZOT_5-1-Et induced phosphorylation of histone H2AX (Ser139) in U-87 MG cells after 2 h treatment as assayed by Western blot. β-actin served as a loading control. DMSO was used as a solvent control.

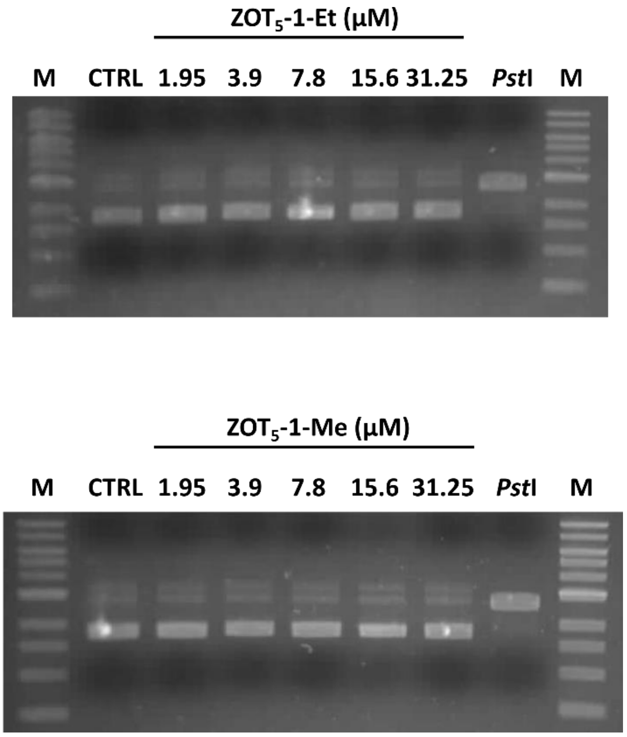

Fig. S4. Raw plasmid relaxation assay gel images from Fig. 5G. ZOT_5-1-Me and ZOT_5-1-Et do not induce DNA breaks in isolated supercoiled plasmid as determined by plasmid relaxation assay following 24 h treatment. Lane M – DNA ladder; CTRL – negative control, supercoiled plasmid, *Pst*I – positive linear control (plasmid incubated with *Pst*I restriction enzyme for the induction of DNA double-strand break.

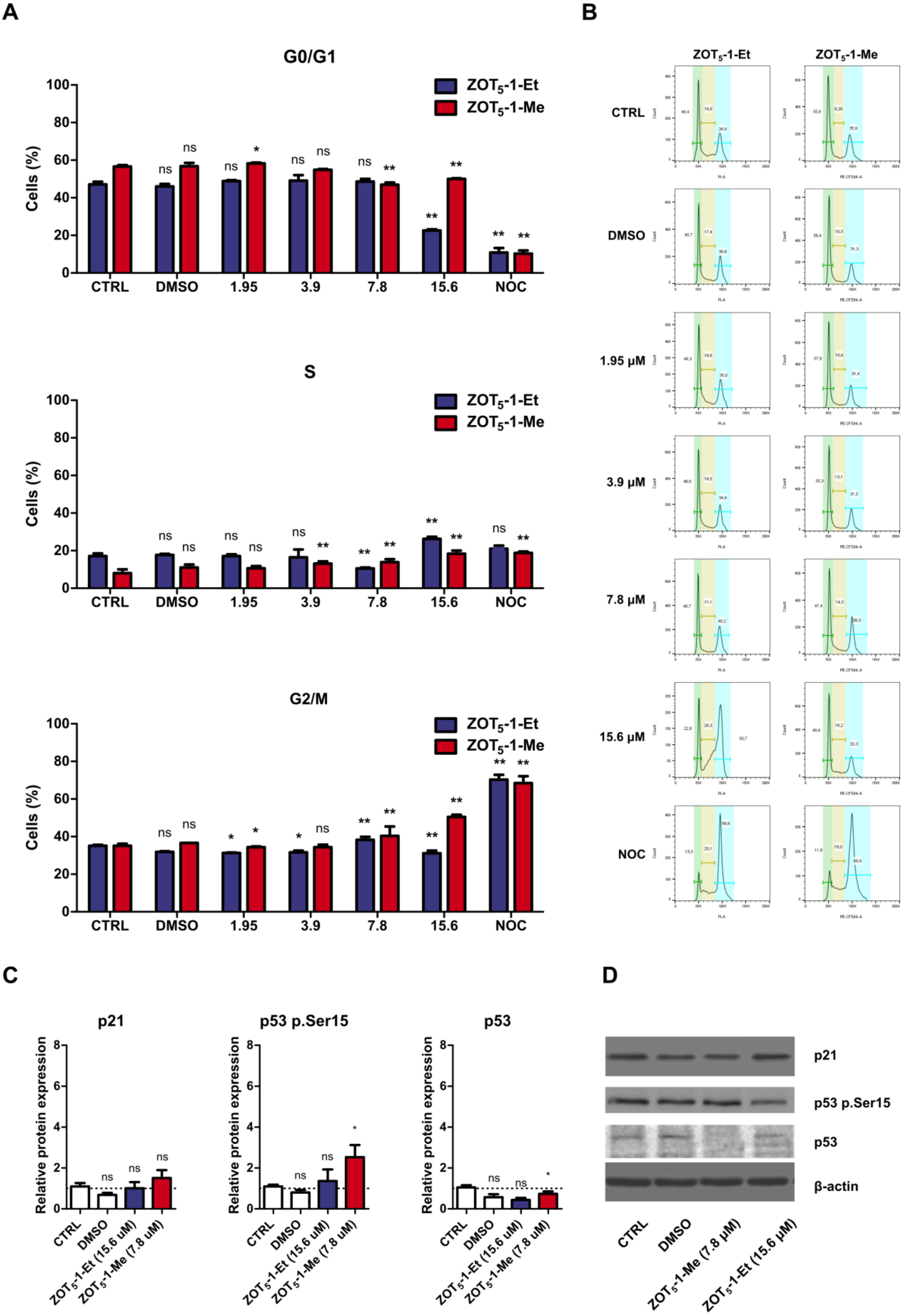

Fig. S5. ZOT5-1-Me and ZOT5-1-Et induced cell cycle arrest in U-87 MG cells. (**A, B**) ZOT5-1-Me and ZOT5-1-Et induced cell cycle arrest in U-87 MG cells after 24 h treatment as determined by PI staining

followed by FACS analysis. Representative FACS histograms are presented. Nocodazole (NOC, 200 ng/mL, 18 h) was used as a positive control of G2/M arrest. Representative FACS dot plots after 24 h treatment with ZOTs are presented. (**D, E**) ZOT_5-1-Me and ZOT_5-1-Et evoked changes to proteins regulating cell cycle, including phosphorylation of p53 (Ser15), in U-87 MG cells after 24 h treatment as evaluated by Western blot (n = 4). The intensity of bands corresponding to proteins was analysed by densitometry. The results are shown as the fold change of proteins levels of treated cells vs. control cells (CTRL). β-actin served as loading control. Representative Western blot images are presented. DMSO was used as solvent control. Results are presented as bar plots with mean ± SEM; *$p < 0.05$; **$p < 0.01$; ***$p < 0.001$, *ns* – not statistically significant.

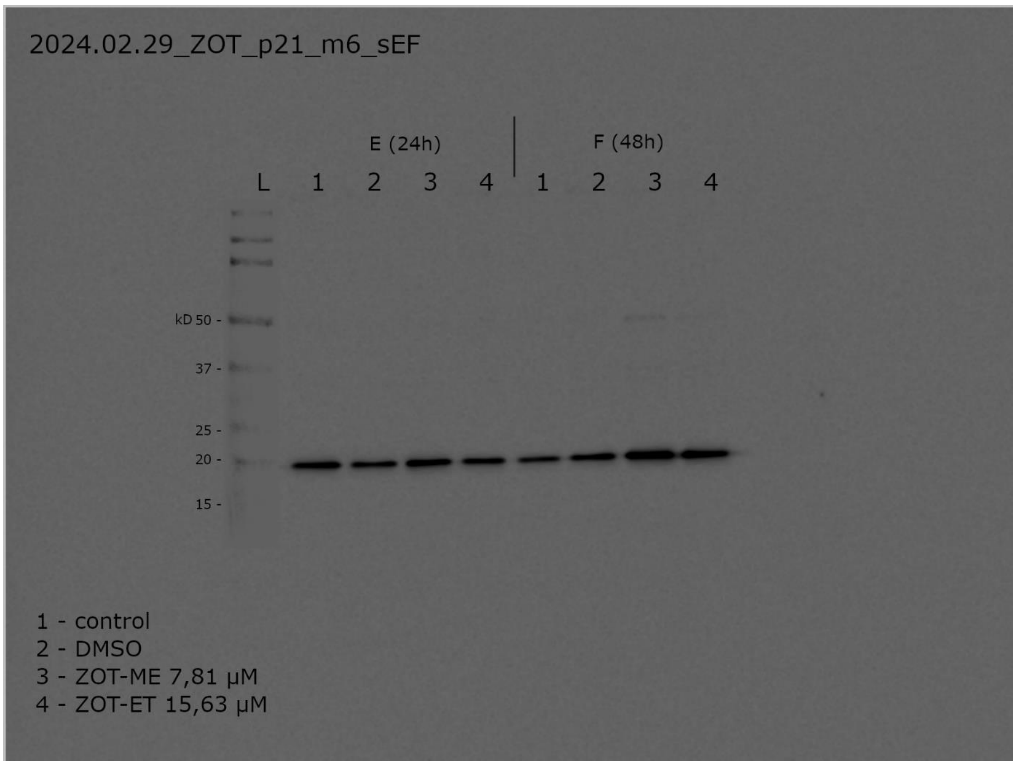
2024.02.29_ZOT_p21_m6_sEF
E (24h)
F (48h)
L 1 2 3 4 1 2 3 4
kD 50 -
37 -
25 -
20 -
15 -
1 - control
2 - DMSO
3 - ZOT-ME 7,81 µM
4 - ZOT-ET 15,63 µM

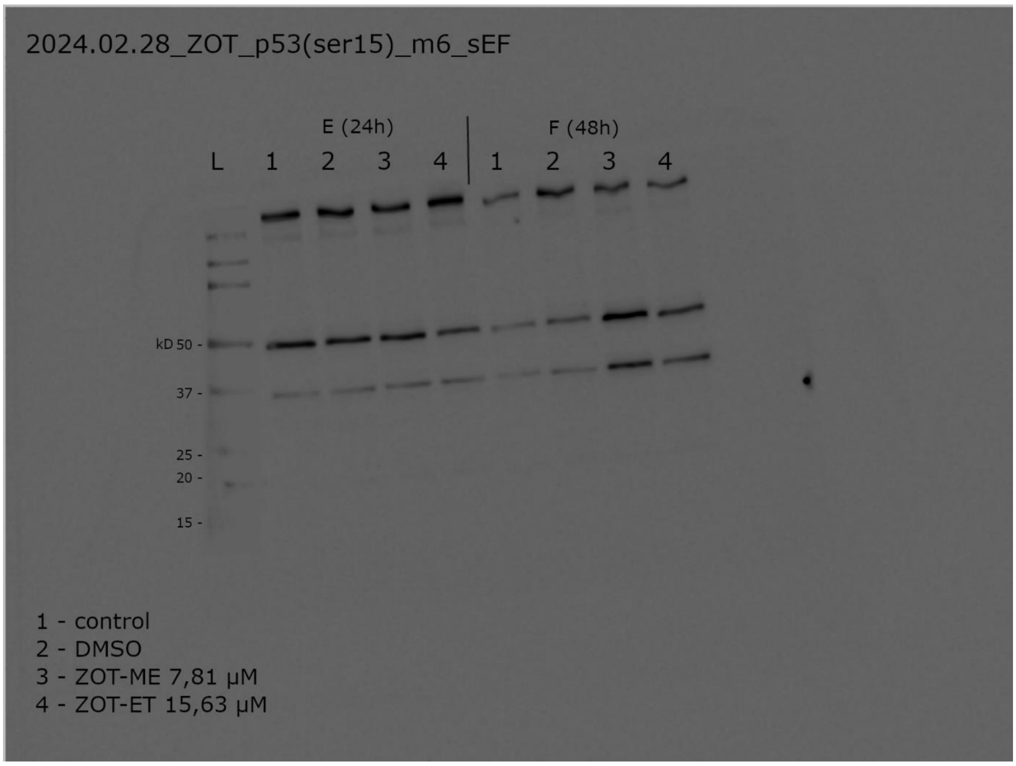
2024.02.28_ZOT_p53(ser15)_m6_sEF
E (24h)
F (48h)
L 1 2 3 4 1 2 3 4
kD 50 -
37 -
25 -
20 -
15 -
1 - control
2 - DMSO
3 - ZOT-ME 7,81 µM
4 - ZOT-ET 15,63 µM

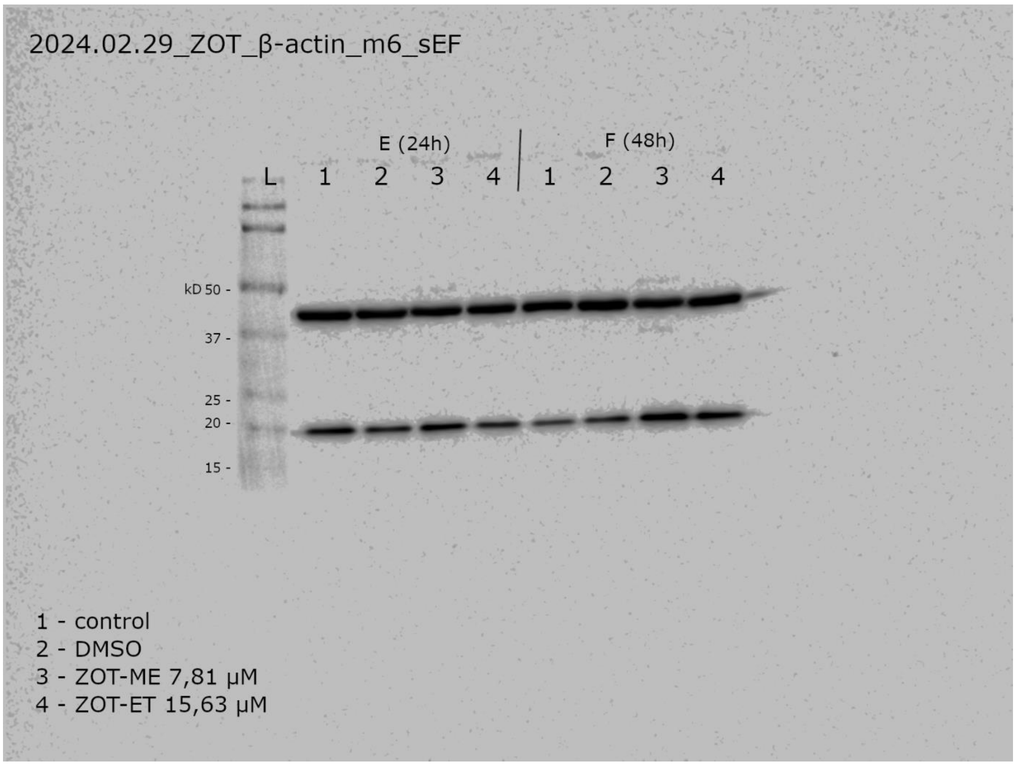

2024.02.29_ZOT_β-actin_m6_sEF
E (24h)
F (48h)
L 1 2 3 4 1 2 3 4
kD 50 -
37 -
25 -
20 -
15 -
1 - control
2 - DMSO
3 - ZOT-ME 7,81 µM
4 - ZOT-ET 15,63 µM

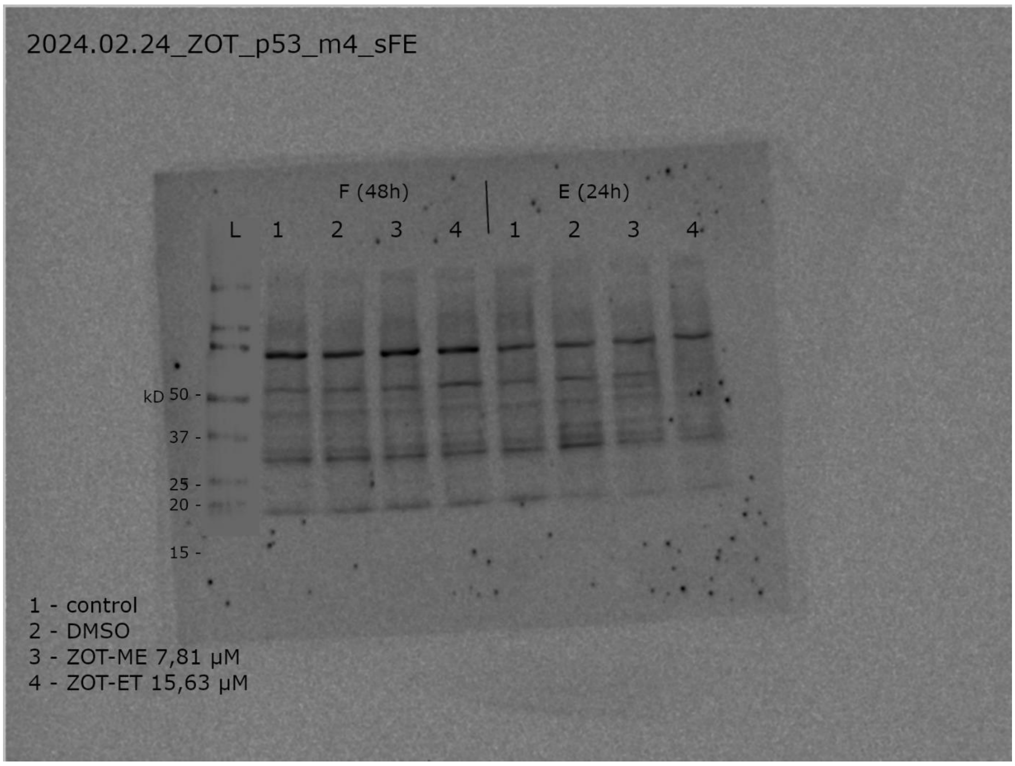

2024.02.24_ZOT_p53_m4_sFE
F (48h)
E (24h)
L 1 2 3 4 1 2 3 4
kD 50 -
37 -
25 -
20 -
15 -
1 - control
2 - DMSO
3 - ZOT-ME 7,81 µM
4 - ZOT-ET 15,63 µM

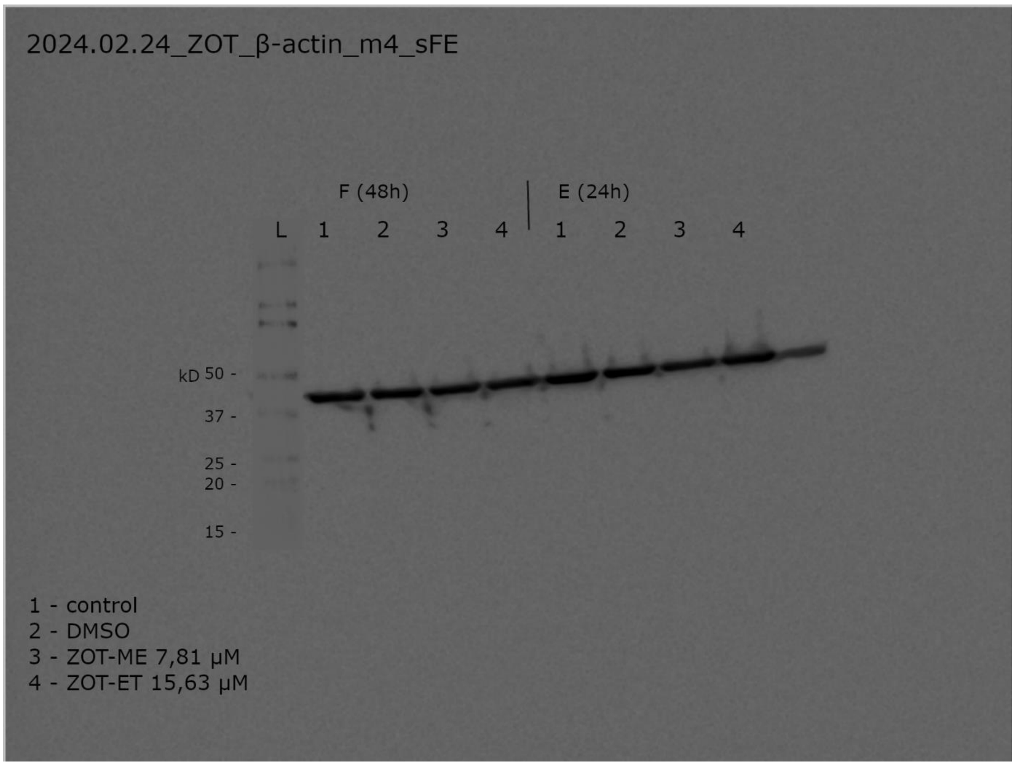

Figure S6. Raw Western blot membranes images from Fig. 6E. ZOT_5-1-Me and ZOT_5-1-Et evoked changes to proteins regulating cell cycle, including p21 and phosphorylation of p53 (Ser15), in U-87 MG cells after 48 h treatment as evaluated by Western blot. β-actin served as a loading control. DMSO was used as a solvent control.
